# Potential Anticancer Lipoxygenase Inhibitors from the Red Sea-Derived Brown Algae *Sargassum cinereum*: An In-Silico-Supported In-Vitro Study

**DOI:** 10.3390/antibiotics10040416

**Published:** 2021-04-10

**Authors:** Sami I. Alzarea, Abeer H. Elmaidomy, Hani Saber, Arafa Musa, Mohammad M. Al-Sanea, Ehab M. Mostafa, Omnia Magdy Hendawy, Khayrya A. Youssif, Abdullah S. Alanazi, Metab Alharbi, Ahmed M. Sayed, Usama Ramadan Abdelmohsen

**Affiliations:** 1 Department of Pharmacology, College of Pharmacy, Jouf University, Sakaka 72341, Aljouf, Saudi Arabia; samisz@ju.edu.sa (S.I.A.); omnia_mmh@yahoo.com (O.M.H.); 2 Department of Pharmacognosy, Faculty of Pharmacy, Beni-Suef University, Beni-Suef 62511, Egypt; abeerabdelhakium@yahoo.com; 3 Department of Botany and Microbiology, Faculty of Science, South Valley University, Qena 83523, Egypt; hani.saber@sci.svu.edu.eg; 4 Department of Pharmacognosy, College of Pharmacy, Jouf University, Sakaka 72341, Saudi Arabia; akmusa@ju.edu.sa (A.M.); Ehabm_y@yahoo.com (E.M.M.); 5 Department of Pharmacognosy, Faculty of Pharmacy, Al-Azhar University, Cairo 11371, Egypt; 6 Department of Pharmaceutical Chemistry, College of Pharmacy, Jouf University, Sakaka 72341, Saudi Arabia; mohmah80@gmail.com; 7 Clinical Pharmacology Department, Faculty of Medicine, Beni-Suef University, Beni-Suef 62511, Egypt; 8 Department of Pharmacognosy, Faculty of Pharmacy, Modern University for Technology and Information, Cairo 11371, Egypt; khayrya.youssif@gmail.com; 9 Department of Clinical Pharmacy, College of Pharmacy, Jouf University, Sakaka 72341, Aljouf, Saudi Arabia; asdalananzi@ju.edu.sa; 10 Department of Pharmacology and Toxicology, College of Pharmacy, King Saud University, P.O. Box 2457, Riyadh 11451, Saudi Arabia; mesalharbi@ksu.edu.sa; 11 Department of Pharmacognosy, Faculty of Pharmacy, Nahda University, Beni-Suef 62513, Egypt; 12 Department of Pharmacognosy, Faculty of Pharmacy, Minia University, Minia 61519, Egypt; 13 Department of Pharmacognosy, Faculty of Pharmacy, Deraya University, 7 Universities Zone, New Minia 61111, Egypt; 14 Health Sciences Research Unit, Jouf university, Sakaka 72341, Aljouf, Saudi Arabia

**Keywords:** *Sargassum cinereum*, metabolic profiling, aryl cresols, docking, 5-LOX, 15-LOX, virtual screening, in silico

## Abstract

LC-MS-assisted metabolomic profiling of the Red Sea-derived brown algae *Sargassum cinereum* “Sargassaceae” dereplicated eleven compounds **1**–**11**. Further phytochemical investigation afforded two new aryl cresol **12**–**13**, along with eight known compounds **14**–**21**. Both new metabolites, along with **19,** showed moderate in vitro antiproliferative activity against HepG2, MCF-7, and Caco-2. Pharmacophore-based virtual screening suggested both 5-LOX and 15-LOX as the most probable target linked to their observed antiproliferative activity. The in vitro enzyme assays revealed **12** and **13** were able to inhibit 5-LOX more preferentially than 15-LOX, while **19** showed a convergent inhibitory activity toward both enzymes. Further in-depth in silico investigation revealed the molecular interactions inside both enzymes’ active sites and explained the varying inhibitory activity for **12** and **13** toward 5-LOX and 15-LOX.

## 1. Introduction

Worldwide, the macroalgal genus *Sargassum* C. Agardh (1820) includes over 537 species, as well as 426 infra-specific names [1]. At present, 361 of the species names have been flagged as accepted taxonomically based on the recorded literature under the species name [1]. *Sargassum* is a cosmopolitan brown algal genus inhabiting temperate subtropical and tropical marine environments, which is identified by non-filamentous thallus with a holdfast that arms to form many central axes [2]. They have specific leaves, receptacles, and vesicles, which are located on the axes near the leaves hold the algal structure upright when submerged [3].

*Sargassum* species are a nutritious and valuable source of bioactive compounds like vitamins, carotenoids, dietary fibers, proteins, and minerals [4]. Additionally, many biologically active compounds, such as terpenoids, flavonoids, sterols, sulfated polysaccharides, polyphenols, sargaquinoic acids, sargachromenol, pheophytin, were separated from different *Sargassum* species [4]. These isolated compounds exhibit distinct biological activities like analgesic, anti-inflammatory, antioxidant, neuroprotective, antimicrobial, antitumor, fibrinolytic, immune-modulatory, anti-coagulant, hepatoprotective, antiviral activity. Therefore, *Sargassum* species have considerable potential to be utilized in pharmaceutical and nutraceutical industries [4,5].

According to the literature, eicosanoids were formed from arachidonic acid (AA) oxidation cascade, which has been linked to pathogenesis for a number of human diseases, including cancer. Nowadays, there is enough evidence supporting their significant role in tumorigenesis and metastases [6,7,8,9].

Although most consideration has focused on prostaglandins (PGs) and another cyclooxygenase (COX)-derived metabolites. There is a growing evidence suggests that lipoxygenases (LOXs)-catalyzed products, such as leukotrienes (LTs), also have profound biological effects on the progression of human cancers [6,7,8,9].

LOXs are a family of non-heme iron-containing enzymes; that catalyze the oxygenation of polyunsaturated fatty acids [9]. Several previous reports on the relationship between LOXs and cancer development support a critical role for 5-lipoxygenase (5-LOX) and 15-lipoxygenase (15-LOX) during the initial stages of prostate breast, colorectal, liver and pancreatic carcinogenesis [6,7,8]. Consequently, using LOXs inhibitors has been shown a vital effect on suppressing the growth of these tumor cells [6,7,8].

In the present study, metabolomic profiling and phytochemical investigation of *S. cinereum* were carried out using liquid chromatography high-resolution mass spectrometry (LC–HRESIMS). Subsequently, unreported hits were isolated along with other major components. The antiproliferative activity of the isolated compounds was tested in vitro against breast Michigan Cancer Foundation-7 (MCF-7), hepatic G2 (HepG2), and colorectal adenocarcinoma-2 (Caco-2) cancer cell lines. Since LOXs have a role in the viability of tumor cells [6,8], A number of isolated compounds were assayed for their 5-LOX and 15-LOX inhibitory activities depending on a prior pharmacophore-based virtual screening. Docking and dynamic studies were conducted to determine the interactions of these compounds inside the enzymes’ active sites.

## 2. Results and Discussion

### 2.1. Chemical Dereplication of S. cinereum

**Figure 1:**
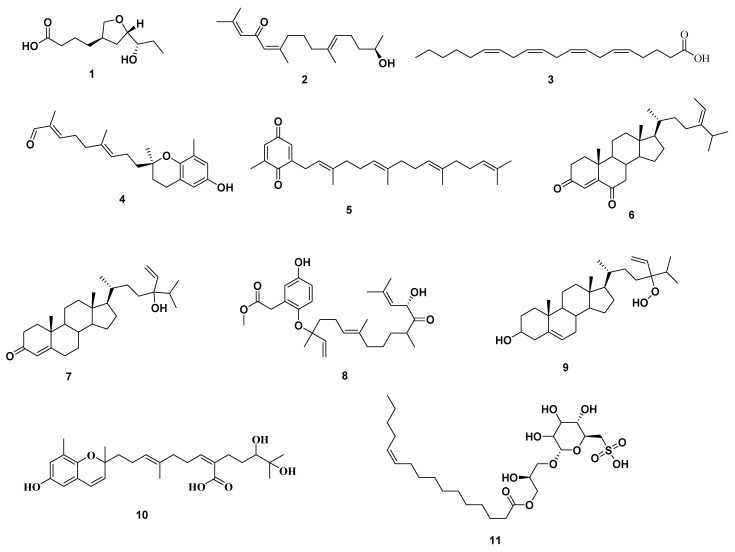
Dereplicated metabolites from liquid chromatography-high resolution electrospray ionization mass spectrometry (LC-HRESIMS) analysis of *S. cinereum.*

Metabolomic profiling of *S. cinereum* alcoholic crude extract, dereplicated eleven compounds, using LC–HRESIMS. The identified metabolites **1**–**11** belonged to different chemical classes, including tetrahydrofuran, hydroquinone, plastoquinone, sterols, meroditerpenoids, and sulfoglycolipid ( Figure 1, Table S1, Figures S1 and S2).

Analysis of *S. cinereum* crude extract led to a putative identification of several hits (Figure 1). The molecular ion mass peaks at *m*/*z* 215.1283 and 277.2162 [M − H]^+^, for the predicted molecular formulas C_11_H_20_O_4_ and C_18_H_30_O_2_ gave hits of (5R,7S,8S)-communiol A **1**, and hedaol A **2**, respectively, that were previously isolated from *Sargassum* spp [10,11]. The mass ion peaks at *m*/*z* 307.2624 and 343.2276 correspond to the suggested molecular formulas C_20_H_34_O_2,_ and C_22_H_30_O_3_ [M+H]+ fit a fatty acid, and hydroquinone anti-inflammatory derivative compound arachidonic acid **3**, and sargachromanol A **4**, that was previously isolated from *Sargassum pallidum*, and *Sargassum siliquastrum,* respectively [12,13]. The ion mass peaks at *m*/*z* 395.2950, 425.3420, 427.3576, and 487.3060 [M + H]^+^ for the predicted molecular formulas C_27_H_38_O_2_, C_29_H_44_O_2_, C_29_H_46_O_2_, and C_29_H_42_O_6_ gave hits of the antiviral plastoquinones 2-geranylgeranyl-6-methylbenzoquinone **5**, which was isolated from *Sargassum micracanthum* [14], the anticancer steroidal nucleus of 24-ethylcholesta-4,24(28)-dien-3,6-dione **6**, saringosterone **7**, which were isolated from *Sargassum carpophyllum,* and *Sargassum asperfolium,* respectively [15,16], and the antioxidant meroditerpenoids of nahocol A **8**, which were isolated from *Sargassum siliquastrum* [17]. Two major ion peaks with the *m*/*z* values of 445.3682 and 459.2749 [M + H]^+^ with molecular formulas C_29_H_48_O_3_ and C_27_H_38_O_6_ were detected and dereplicated as 24xi-hydroperoxy-24-vinylcholesterol **9** and sargathunbergol A **10**, respectively, which were isolated earlier from *Sargassum carpophyllum,* and *Sargassum thunbergii,* respectively [15,18].

In addition, the mass ion peaks at *m*/*z* 553.2681 [M − H]^+^, for the predicted molecular formula C_25_H_46_O_11_S was dereplicated sulfoglycolipid derivative 1-*O*-(11-Hexadecenoyl)-3-*O*-(6′-sulfo-*α*-d-quinovopyranosyl) glycerol **11**, which was previously detected in *Sargassum hemiphyllum* (Figure 1) [19].

### 2.2. Phytochemical Investigation of S. cinereum

Based on the physicochemical and chromatographic properties, the spectral analyses from UV, ^1^H, and DEPT-Q NMR, as well as comparisons with the literature and some authentic samples, the crude alcoholic extract of *S. cinereum* afforded the new aryl cresol **12**–**13**, along with the known *O*-cresol **14** [20], *m*-cresol **15** [21]. Additionally, arachidonic acid **16** [22], eicosenoic acid **17** [22], 1-*O*-arachidonyl-glycerol **18** [23], 1-*O*-arachidonyl-3-*O*-(*α*-d-glucopyranosyl) glycerol **19** [23], 7-β-methyl androstenol **20** [24], and 1-deoxy-β-d-psicosofuranose **21** [25], were identified (Figure 2). All characterized compounds **14** and **15** were isolated herein for the first time from the genus *Sargassum* (Figure 2, Figures S3–S28).

Analysis of the HRESIMS, 1D and 2D NMR data of compounds **12**–**13** suggested a possible plastoquinones core scaffold [11]. The HRESIMS data for compound **12** showed an adduct pseudo molecular ion peak at *m*/*z* 314.2607 [M + H]^+^ (calc. for C_22_H_34_O, 314.2604), suggesting 7 degrees of unsaturation. The ^1^H and DEPT-Q ^13^C NMR data (Table 1 and Figures S3 and S4), along with the heteronuclear single quantum correlation experiment (HSQC) data (Figure S5), suggested six characteristic resonances appeared: three aromatic methine groups at *δ*_H_ 6.68 (1H, s) *δ*_C_ 116.0, *δ*_H_ 6.98 (1H, d, *J* = 8.0 *δ*_C_ 123.6, *δ*_H_ 7.13 (1H,d, *J* = 8.0) *δ*_C_ 123.1, three quaternary carbons at *δ*_C_ 153.8, 140.6, and 134.5, and one methyl group at *δ*_H_ 1.34 (1H, s) *δ*_C_ 29.8, suggesting the characteristic core structure for a tri-substituted benzene unit [11].

NMR data also showed eight aliphatic methylene groups at *δ*_H_ 1.20–2.8 *δ*_C_ 20.5–33.7 (Table 1), three olefinic methine groups at *δ*_H_ 5.31–5.35 (6H, m) *δ*_C_ 127.9–129.4. These signals are suggestive characteristics for 4,7,11-pentadecenyl moiety, where the heteronuclear multiple-bond correlation (HMBC) experiment of **12** (Figure 3) confirmed the position of the three olefinic methine groups at 4,7,11 of the alkene side-chain. Moreover, the HMBC experiment showed the ^3^ *J*-HMBC correlation of the proton H-1′ *δ*_H_ 2.26 (*δ*_C_ 33.4) with the quaternary carbonyl carbon C-4′ (*δ*_C_ 134.5). Accordingly, compound **12** was identified as 4-(1-(4,7,11-pentadecenyl)-*o*-cresol. 

The molecular formula of compound **13** was identical to that of **12** based on HRESIMS (C_22_H_34_O). The ^1^H and ^13^C NMR data was also very close to those of compound **12** for the 4,7,11-pentadecenyl moiety but differed in the resonated chemical shifts of the aromatic attached methyl group of the core tri-substituted benzene unit (Table 1). Comparing the DEPT-Q ^13^C NMR data of compound **13** with those of **12** showed a downfield shifting of carbons C-7 (Δ*δ*_C_ + 2.1), compared with those of compound **12** (Table 1). This suggested a positional difference of the location of the aromatic attached methyl group in the tri-substituted benzene unit versus **12** (Table 1 and Supplementary File 1(Figure S2 and S8–S12)). The assignment of the location of the aromatic attached methyl group in **13** was aided by the HMBC experiment. A ^3^ *J*-HMBC correlation (Figure 4) of compound **13** proton H-7 *δ*_H_ 1.23 (*δ*_C_ 31.9) with the quaternary carbonyl carbon C-4 (*δ*_C_ 134.5) and a ^4^ *J*-HMBC correlation of the proton H-7 *δ*_H_ 1.23 (*δ*_C_ 31.9) with the methylene carbon C-1′ (*δ*_C_ 33.7) confirmed the meta-location of an aromatic attached methyl group at the cresol moiety. Accordingly, compound **13** was identified as 4-(1-(4,7,11-pentadecenyl)-*m*-cresol.

### 2.3. Antiproliferative Activity of the Isolated Metabolites

The isolated compounds **12**–**21** were in vitro screened for their antiproliferative activity against hepatic, breast, and colorectal carcinoma cell lines (HepG2, MCF-7, and Caco-2, respectively) using the sulforhodamine B (SRB) assay. Results showed that compounds **12**, **13**, and **19** were able to inhibit the growth of all tested cell lines moderately with IC_50_ values ranged from 11.2 ± 0.6 to 21.6 ± 1.3 µM (Table 2).

### 2.4. Virtual Screening-based Target Identification

Characterization of the biological target for a certain molecule is a true challenge. However, the continuous development of in silico tools, including molecular modeling and virtual screening, has significantly improved the success rate of finding suitable molecular targets. Many online target identification platforms are currently available, and their search protocols are either structural-based or ligand-based. PharmMapper is one of these online platforms that can screen and suggest the most probable protein targets of a query molecule based on its pharmacophore model [26]. The basic principle of pharmacophore-based screening is that the binding of certain molecules with their protein targets is mainly determined by key pharmacophore maps (i.e., spatial arrangement of structural features). Thus, molecules that shapes are able to fit with these pharmacophore maps have the highest probability to bind the same protein target. Consequently, PharmMapper was used to propose a proper protein target for compounds **12**, **13** and **19**. 5-LOX and 15-LOX were found to be the top-scoring hits for these metabolites. As discussed in the introduction, these enzymes have been shown a direct link to the development of many cancers, e.g., breast, colorectal, liver, skin cancers [6,7,8,27,28,29,30]. Herein, compounds **12**, **13** and **19** showed considerable inhibitory activity towards the human breast, colorectal, and liver cancer cell lines, and hence, they were selected for further in vitro and in silico validations against 5-LOX and 15-LOX.

### 2.5. LOX Inhibition Assay

To validate the preliminary virtual screening prediction, compounds **12**, **13** and **19** were assayed for their 5-LOX and 15-LOX inhibitory activities. Interestingly the three compounds achieved potent enzyme inhibition toward 5-LOX (IC_50_ 1.3 ± 0.1 to 2.1 ± 0.4 µM, Table 3). However, their activity against 15-LOX was weaker, particularly compounds **12** and **13** (IC_50_ 25.3 ± 0.4 and 23.6 ± 0.3 µM, respectively) that were more selective for 5-LOX (Table 3).

Moreover, they showed inhibitory constants (*K*_i_) ranged from 0.7 ± 0.2 to 17.4 ± 0.2 µM (Table 3), and these values were most agree with the competitive inhibition of both enzymes [31].

The results of enzyme inhibition assay were also correlated with those of the antiproliferative ones for HepG2 and MCF-7, and Caco-2. Overexpression of 5-LOX has been reported in breast, liver and colorectal cancers [27,28,29]. Furthermore, 15-LOX has been reported to be overexpressed in a number of tumors like prostate and breast cancers. Hence, these enzymes can be considered promising targets for cancer therapy.

### 2.6. Molecular Docking and Dynamic Simulation

**Figure 2:**
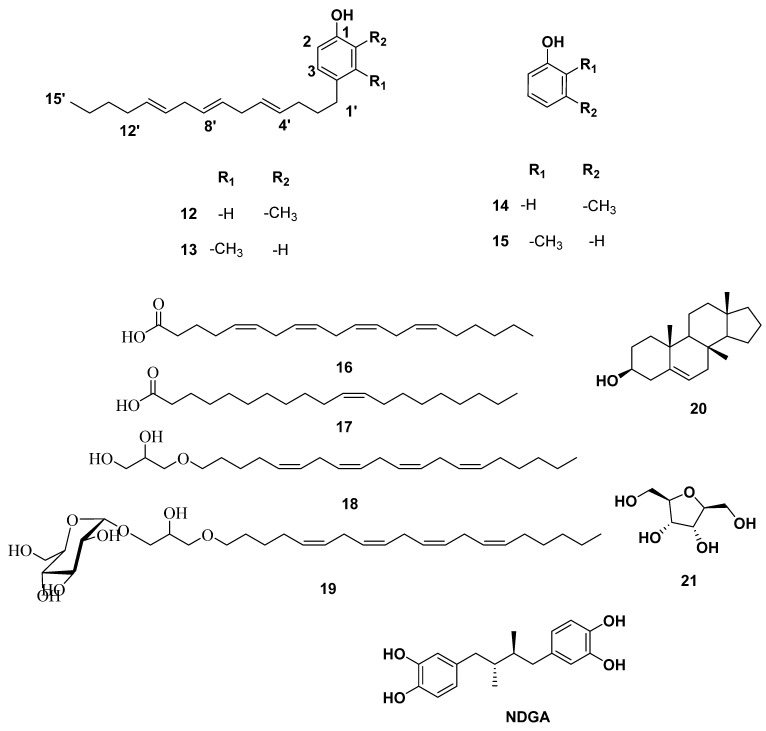
Structures of compounds isolated from *S. cinereum* **12**–**21** together with 5-lipoxygenase (5-LOX) and 15-LOX’s co-crystallized ligands AA **16** and nordihydroguaiaretic acid (NDGA).

**Figure 3:**
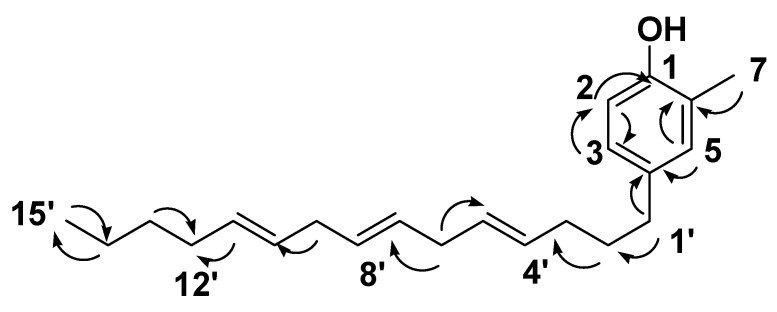
Selected heteronuclear multiple-bond correlation (**HMBC)** (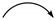) correlations of compound **12.**

**Figure 4:**
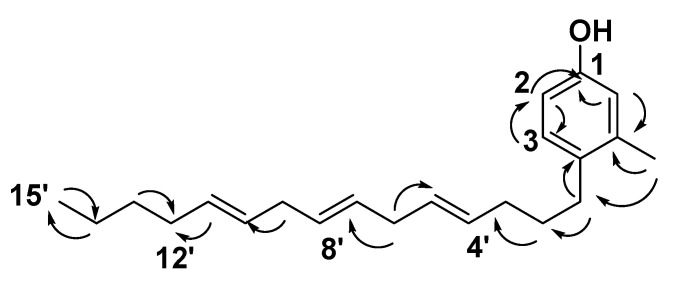
Selected **HMBC** (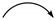) correlations of compound **13.**

**Figure 5:**
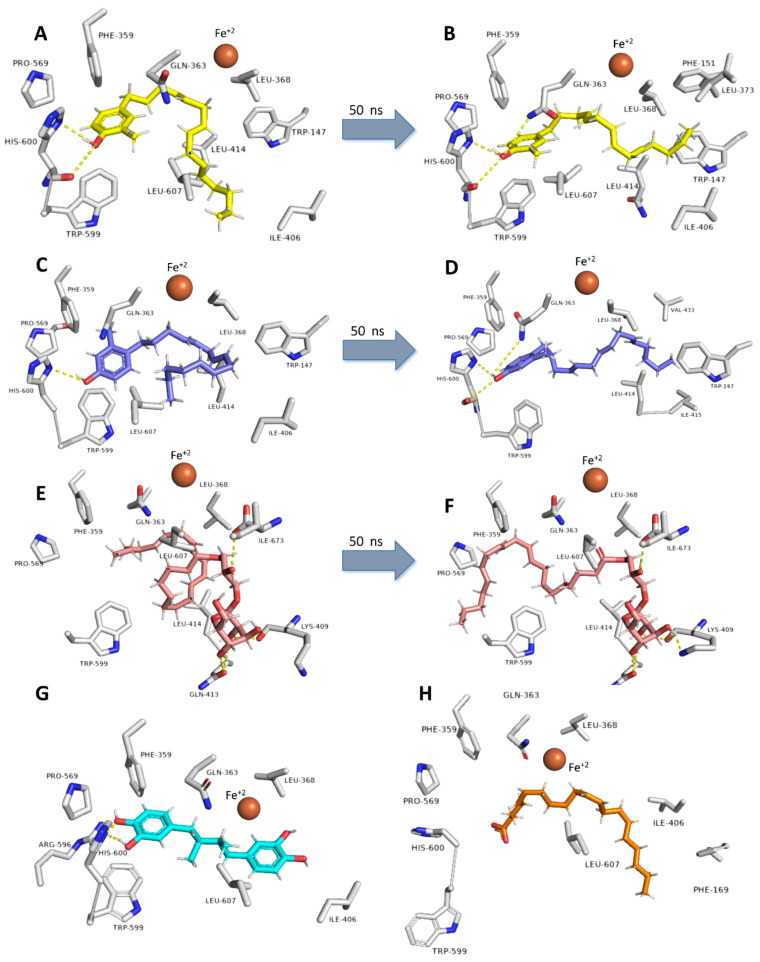
Binding modes of compounds **12**, **13**, and **19** inside 5-LOX’s active site. (**A**,**C**,**E**) Their binding modes upon docking. (**B**,**D**,**F**) Their binding modes over 50 ns MDS. (**G**,**H**) Binding modes of the co-crystalized ligands AA and NDGA.

5-LOX has a hydrophobic active site [9] that harbors a catalytic iron (Fe^+2^), and such hydrophobicity is essential to allow efficient binding with the hydrophobic substrate arachidonic acid (AA) (Figure 2) [9]. Compounds **12**, **13**, and **19** have extended unsaturated hydrophobic side chains that resemble AA (Figure 5).

Molecular docking experiments revealed that these compounds could bind with the 5-LOX’s active site efficiently, with binding scores ranged from −8.9 to −9.3 kcal/mol (Figure 5), and their bindings were even better than the co-crystalized ligands (Table 3). Additionally, the phenolic moiety of both compounds was involved in H-bonding with HIS-600, similarly to the co-crystallized redox-type inhibitor, nordihydroguaiaretic acid (NDGA) (Figure 5).

Compounds 12 and 13s hydrophobic side chains were able to adapt themselves inside the hydrophobic U-shaped active site, where they took convergent orientations but slightly different from that of AA (Figure 5). LEU-368, ILE-406, LEU-414, and LEU-607 were the main amino acid residues involved in the hydrophobic interactions with their side chains, while PHE-359, TRP-599, and PRO-569 interacted with their aromatic moieties. The binding mode of compound 19 was quite different, where its polar carbohydrate head interacted with LYS-409, GLN-413, and ILE-673 through four strong hydrogen bonds (<2.5 Å), while it is hydrophobic tail interacted with LEU-368, LEU-414, TRP-599, and LEU-607 (Figure 5). Subsequent molecular dynamic simulation (MDS) experiments (50 ns) revealed that the three compounds 12, 13, and 19 were able to stabilize the enzyme’s active site.

**Figure 6:**
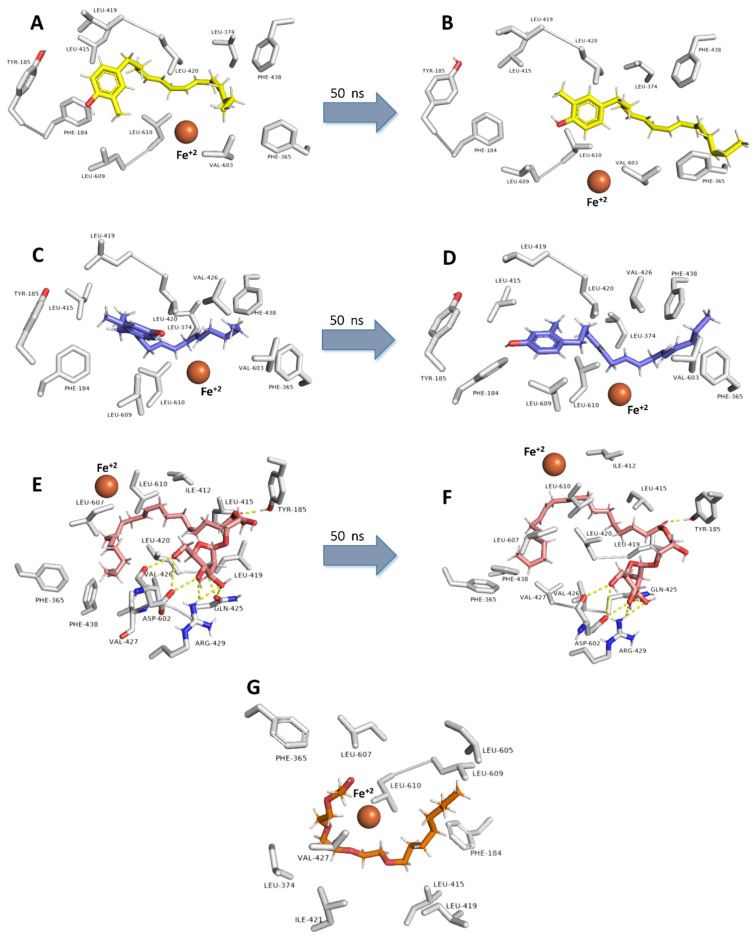
Binding modes of compounds **12**, **13**, and **19** inside 15-LOX’s active site. (**A**,**C**,**E**) Their binding modes upon docking. (**B**,**D**,**F**) Their binding modes over 50 ns MDS. (**G**) Binding mode of the co-crystalized ligands AA.

Compounds 12 and 13s positions remained to change over the first 32.4 ns (RMSD~3.4 Å). Afterward, they became stable till the end of the simulation (average RMSD values of 2.67 and 2.59 Å, respectively), where their extended hydrocarbon chains became more relaxed and straight (Figure 6). The H-bonds between their phenolic group and HIS-600 remained unchanged throughout the MDS. Starting from 22.6 ns, GLN-363’s side-chain became also involved in H-bonding with the phenolic group of both compounds (Figure 6). Additionally, compound 12 s tail remained imbedded inside a hydrophobic pocket consists of the side chains of TRP-147, PHE-151, LEU-368, LEU-373, and LEU-414, while compound 13 s tail settled inside another hydrophobic pocket consists of TRP-147, LEU-414, ILE-415, and VAL-433 (Figure 6).

**Figure 7:**
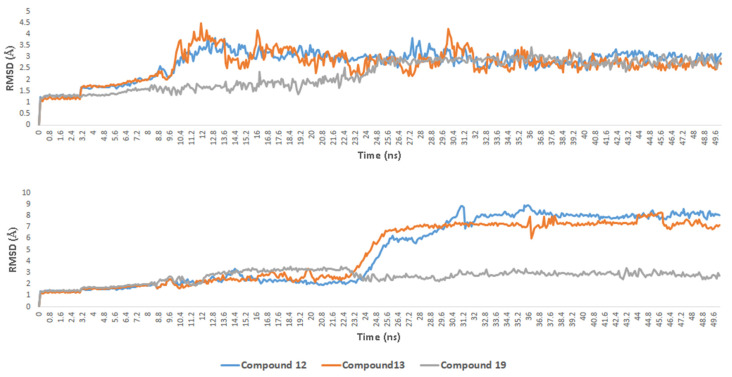
RMSDs of compounds **12**, **13** and **19** inside 5-LOX and 15-LOX’s active sites over 50 ns of molecular dynamic simulations (MDS).

Similarly, the hydrophobic part of compound 19 was compacted at the beginning of MDS and gradually become more extended till stabilization at 25.4ns (RMSD = 2.75 Å), where PHE-359, PRO-569, and TRP-599 became involved in hydrophobic interactions with the molecule’s tail. Furthermore, the side-chain of LYS-409 became involved in an additional H-bonding with the molecule’s hydrophilic carbohydrate part (Figure 6). Further binding free energy calculations (Δ*G*_FEP_ and Δ*G*_K_*_DEEP_*) revealed that compounds 12, 13, and 19 got higher binding free energy values than that of the co-crystalized ligands (Figure 7) and were in good accordance with the in vitro enzyme inhibition (Figure 7).

On the other hand, docking scores of the three compounds against 15-LOX were significantly lower, particularly for compounds 12 and 13 (Figure 7) that showed only hydrophobic interactions (with PHE-184, TYR-185, PHE-365, LEU-374, LEU-415, LEU-419, LEU-420, VAL-603, LEU-609, and LEU-610) inside the enzyme’s active site. Upon MDS (Figure 7), both compounds showed unstable positioning inside the 15-LOX active site, where the surrounding hydrophobic amino acid residues were able to keep them in position till ~23.4 ns (RMSD~3.5 Å). Afterward, their position inside the active site began to change dramatically, and their RMSDs reached reach about 7.1 Å and remained to fluctuate till the end of MDS with gross averages of 8.1 and 7.5 Å, respectively, over the 50ns of simulation. This obvious instability of compounds 12 and 13 reflected on their binding free energies and in vitro inhibitory activities that were significantly lower than 5-LOX (Figure 7).

Such instability was not the case with compound 19, where the hydrophilic carbohydrate moiety was able to form a network of H-bonds (7 H-bonds) with TYR-185, GLN-425, ARG-429, and ASP-602. Furthermore, these hydrophilic interactions remained unchanged over the course of MDS, and thus compound 20 s RMSD was at equilibrium (~2.6 Å) to the end of MDS. Such structural and dynamic information can explain the convergent inhibitory activity of compound 19 against both 5-LOX and 15-LOX (Figure 7) and the moderate selective inhibition of 5-LOX by compounds 12 and 13 over 15-LOX.

## 3. Materials and Methods

### 3.1. Algae Material

The marine algae *S. cinereum* was collected during January 2020 along the shore of the Red Sea in Hurghada, Egypt. The samples were collected in sterilized polyethylene bags and kept in an icebox for transportation to the laboratory. Samples were washed thoroughly with sterile distilled water to remove any associated debris. A voucher specimen (2020-BuPD 55) was deposited at the Department of Pharmacognosy, Faculty of Pharmacy, Beni-Suef University, Egypt.

### 3.2. Chemicals and Reagents

The solvents used in this work included *n*-hexane (*n*-hex., boiling point b.p. 60–80 °C), dichloromethane (DCM), ethyl acetate (EtOAc), *n*-butanol (*n*-but.), and methanol (MeOH) were purchased from El-Nasr Company for Pharmaceuticals and Chemicals (Egypt). High-performance liquid chromatography (HPLC) and deuterated solvents used for chromatographic and spectroscopic analyses were purchased from Sigma-Aldrich (Saint Louis, MO, USA), including HPLC–methanol, HPLC–water, HPLC–acetonitrile, deuterium oxide (D_2_O), methanol (CD_3_OD), and dimethyl sulfoxide (DMSO-*d*_6_). Column chromatography (CC) was performed using silica gel 60 (63–200 μm, E. Merck, Sigma-Aldrich), and Sephadex LH-20 (0.25–0.1 mm, GE Healthcare, Sigma-Aldrich, Steinheim, Germany), while silica gel GF254 for thin-layer chromatography (TLC) (El-Nasr Company for Pharmaceuticals and Chemicals, Egypt) was employed for vacuum liquid chromatography (VLC). Thin-layer chromatography (TLC) was carried out using precoated silica gel 60 GF254 plates (E. Merck, Darmstadt, Germany; 20 × 20 cm, 0.25 mm in thickness). Spots were visualized by spraying with para-anisaldehyde (PAA) reagent (85:5:10:0.5 absolute EtOH:sulfuric acid:G.A.A.:para-anisaldehyde), followed by heating at 110 °C [32]. For the biological study, doxorubicin (Sigma-Aldrich, Germany) was used as a positive control, while the HepG2, MCF-7, and Caco-2 cancer cell lines were obtained from the American Type Culture Collection (ATCC, Rockville, MD, USA; HPACC, Salisbury, UK) and were routinely subcultured twice per week.

### 3.3. Spectral Analyses

Proton ^1^H and distortionless enhancement by polarization transfer-Q (DEPT-Q) ^13^C NMR spectra were recorded at 400 and 100 MHz, respectively. Tetramethylsilane (TMS) was used as an internal standard in deuterium oxide (D_2_O), methanol (CD_3_OD), and dimethyl sulfoxide (DMSO-*d*_6_), using the residual solvent peak (*δ*_H_ = 4.78), (*δ*_H_ = 3.34, 4.78 and *δ*_C_ = 49.9) and (*δ*_H_ = 2.50 and *δ*_C_ = 39.5) as references, respectively. Measurements were performed on a Bruker Advance III 400 MHz with BBFO Smart Probe and a Bruker 400 MHz EON nitrogen-free magnet (Bruker AG, Billerica, MA, USA). Carbon multiplicities were determined using a DEPT-Q experiment. The ultraviolet radiation (UV) spectrum in methanol was obtained using a Shimadzu UV 2401PC spectrophotometer (Shimadzu Corporation—UV-2401PC/UV-2501PC, Kyoto, Japan). Infrared (IR) spectra were measured using a Jasco FTIR 300E infrared spectrophotometer. HRESIMS data were obtained using an Acquity ultra-performance liquid chromatography system coupled to a Synapt G2 HDMS quadrupole time-of-flight hybrid mass spectrometer (Waters, Milford, MA, USA). HPLC chromatographic separations were conducted using an Agilent 1260 Infinity preparative pump (G1361A), Agilent 1260 diode array detector VL (G1315 D), Agilent 1260 Infinity Thermostand column compartment (G1361 A), Agilent 1260 Infinity preparative autosampler (G2260A) and a YMC-Pack ODS-A A-324 column (i.d. 10 × 300 mm, YMC, Kyoto, Japan).

### 3.4. Extraction and Fractionation of Algae Material

*Sargassum cinereum* (0.5 kg) was collected and air-dried in the shade for one month. After drying, the brown algae were finely powdered using an OC-60B/60B grinding machine (60–120 mesh, Henan, China). The finely powdered algae extracted by maceration using 70% methanol (3 L, 3×, seven days each) at room temperature, and concentrated under vacuum at 45 °C using a rotary evaporator (Buchi Rotavapor R-300, Cole-Parmer, Vernon Hills, IL, USA) to afford 75 g crude extract. The dry extract was suspended in 100 mL distilled water (H_2_O) and successively portioned with solvents of different polarities (*n*-Hex., DCM, EtOAc, and *n*-but.). The organic phase in each step separately evaporated under reduced pressure to afford the corresponding fractions I (8.0 g), II (1.5 g), III (1.5 g) and IV (3.0 g), respectively, while the remaining mother liquor was then concentrated down to give the aqueous fraction (V). All resulting fractions were kept at 4 °C for biological and phytochemical investigations.

### 3.5. Metabolomic Analysis Procedure

The crude methanolic extract from *S. cinereum* was prepared at 1 mg/mL for mass spectrometry analysis. The recovered methanolic extract was subjected to metabolic analysis using LC-HRESIMS according to Abdelmohsen et al. 2014 [33]. An Acquity ultra-performance liquid chromatography system connected to a Synapt G2 HDMS quadrupole time-of-flight hybrid mass spectrometer (Waters, Milford, M.A. USA) was used. Positive and negative ESI ionization modes were utilized to carry out the high-resolution mass spectrometry coupled with a spray voltage at 4.5 kV, the capillary temperature at 320 °C, and mass range from *m/z* 150–1500. The MS dataset was processed, and data were extracted using MZmine 2.20 based on the established parameters [22]. Mass ion peaks were detected and accompanied by chromatogram builder and chromatogram deconvolution. The local minimum search algorithm was addressed, and isotopes were also distinguished via the isotopic peaks of grouper. Missing peaks were displayed using the gap-filling peak finder. An adduct search along with a complex search was carried out. The processed data set was next subjected to molecular formula prediction and peak identification. The positive and negative ionization mode data sets from the respective extract were dereplicated against the Dictionary of Natural Products (DNP) databases.

### 3.6. Isolation and Purification of Major Compounds

Fraction I (8 g) was subjected to normal VLC fractionation using silica gel GF_254_ (column 6 × 30 cm, 50 g). Elution was performed using *n*-hex.:EtOAc gradient mixtures in order of increasing polarities (0, 5, 10, 15, 20, 25, 30, 35, 40, 45, 50, 60, 80 and 100%, 500 mL each). The effluents from the column were collected in fractions (100 mL each), and each collected fraction was concentrated and monitored by TLC using the system *n*-hex.:EtOAc 8:2 and PAA reagent. Similar fractions were grouped and concentrated under reduced pressure to provide three subfractions (I_1_–I_3_). Subfraction II_2_ (3.0 g) was further fractionated on silica gel 60 (100 × 1 cm, 50 g). Elution was performed using *n*-hex.:EtOAc gradient mixtures in the order of increasing polarities (0, 1, 2, 3, 4, 5, 6, 7, 8, 9 and 10%, 1 L each, FR 3 mL min^−1^), to afford four sub-subfractions (II_2-1_–II_2-4_). Sub-subfraction II_2-1_ (50 mg) was further fractionated on silica gel 60 (100 × 1 cm, 20 g). Elution was performed using *n*-hex.:EtOAc isocratic mixture (1%, 500 mL, FR 3 mL min^−1^) to afford compound **17** (20 mg). Sub-subfractions II_2-2_, and II_2-4_ (70, 30 mg each) was further fractionated on C-18 RP-HPLC using H_2_O-CH_3_CN (10–60%, 30 min, 5 mL/min) to afford compound **12** (20 mg), **13** (10 mg), **14** (10 mg), **15** (7 mg). Sub-subfraction II_2-3_ (100 mg) was further fractionated on silica gel 60 (100 × 1 cm, 20 g). Elution was performed using *n*-hex.:EtOAc isocratic mixture (5%, 500 mL, FR 3 mL min^−1^) to afford compound **16** (50 mg). Finally, subfraction II_3_ was further fractionated on silica gel 60 (100 × 1 cm, 20 g). Elution was performed using *n*-hex.:EtOAc isocratic mixture (1%, 500 mL, FR 3 mL min^−1^) to afford compound **20** (30 mg). Fraction II (1.5 g) was subjected to normal VLC fractionation on a silica gel (column 6 × 30 cm, 50 g). Elution was performed using DCM:MeOH gradient mixtures in the order of increasing polarities (0, 5, 10, 15, 20, 25, 30, 35, 40, 45, 50, 60, 80 and 100%, 1 L each). The effluents were collected in fractions (100 mL each); each fraction was concentrated and monitored by TLC using the system DCM:MeOH 9.5:0.5 and PAA reagent. Similar fractions were grouped and concentrated under reduced pressure to provide two subfractions (II_1_–II_2_), which were further purified on a Sephadex LH_20_ column (0.25–0.1 mm, 100 × 0.5 cm, 100 g), which eluted with MeOH to afford compound **18** (16 mg), and **19** (6 mg), separately.

Crystallization of fractions IV was performed separately using CH_2_CL_2_ and afforded compounds **21** (2 g).

4-(1-(4,7,11-pentadecenyl)-*o*-cresol (12): Yellow oil; [UV (MeOH) *λ*_max_ (log_ε_) 225 (5.5), 260 (6.0), 300 (4.5) nm; IR υ_max_ (KBr) 3429, 3100, 3000, 1680, 1600, 1475, 1450, 1300, 835, 601 cm^−1^; NMR data; see Table 1; HRESIMS m/z 314.2607 [M + H]^+^ (calc. for C_22_H_34_O, 314.2604).

4-(1-(4,7,11-pentadecenyl)-*m*-cresol (13): Yellow oil; UV (MeOH) λ_max_ (log_ε_) 225 (5.5), 260 (6.0), 300 (4.5) nm; IR υ_max_ (KBr) 3429, 3100, 3000, 1680, 1600, 1475, 1450, 1300, 835, 601 cm^−1^; NMR data; see Table 1; HRESIMS m/z 314.2609 [M + H]^+^ (calc. for C_22_H_34_O, 314.2604).

### 3.7. Antiproliferative Assay

The antiproliferative activity of the isolated compounds **12**–**21** was measured by the sulforhodamine B (SRB) assay as described by Skehan et al. 1990 [34], and Vichai and Kirtikara 2006 [35], on the breast (MCF-7), liver (HepG2) and colorectal (Caco-2) cancer cell lines. Cells were seeded in 96-well microtiter plates at an initial concentration of 3 × 10^3^ cell/well in 150 µL, fresh medium and left for 24 h to attach to the plates. Different concentrations 0, 5, 12.5, 25, 50 µg/mL of the respective compound were added. The plates were incubated for 48 h. The cells were fixed with 50μL cold trichloroacetic acid (10% final concentration) for 1 h at 4 °C. The plates were washed with distilled water (automatic washer Tecan, Neustadt, Germany) and stained with 50 μL 0.4% SRB dissolved in 1% acetic acid for 30 min., at room temperature. Then they were washed with 1% acetic acid and air-dried. The dye was solubilized with 100 μL/well of 10 M Tris base (pH 10.5). The optical density of each well was measured spectrophotometrically at 570 nm using an ELISA microplate reader (Sunrise Tecan reader, Neustadt, Germany). Doxorubicin was used as a positive control. The mean background absorbances were automatically subtracted, and the mean values of each drug concentration were calculated. The experiment was repeated three times, and then the IC_50_ values were calculated.

### 3.8. Lipoxygenase (LOX) Inhibition Assay

The ability of the isolated compounds **12**, **13**, and **19** to inhibit 5-LOX and 15-LOX enzymes (IC_50_ and *K*_i_ values, µM) was determined using human recombinant enzyme assay kits (catalog no 60,402 and 10011263, Cayman Chemical, Ann Arbor, MI, USA) following manufacturer’s specifications [36]. Stock solutions were freshly prepared before use, and buffer solution (0.1 M Tris-HCl, PH, 7.4) was used. 10 µL of each compound were prepared, dissolved in the least amount of DMSO and diluted with the stock solution to be in concentrations of (0.001, 0.1, 1, 5, 10 µM) in a final volume of 210 mL. The kinetic parameters for both 5-LOX and 15-LOX were determined by measuring the increase in absorbance at 238 nm in an Agilent 8453 diode array spectrophotometer (Agilent Technologies, Santa Clara, CA, USA). Substrate concentration was ranged from 5 to 50 µM. Substrate concentrations (5, 10, 20, 30, 40, 50 µM) were monitored in triplicate for each sample [37]. Doxorubicin was used as a positive control.

### 3.9. Docking Study

The crystal structures of both 5-LOX and 15-LOX (PDB: 6N2W and 4NRE) were used for the docking analysis using an AutoDock Vina docking machine [38]. The co-crystallized ligands nordihydroguaiaretic acid (NDGA) and AA were used to determine the binding sites. The ligand to binding site shape matching root means square (RMSD) threshold was set to 2.0 Å. The interaction energies were determined using the Charmm force field (v.1.02) with 10.0 Å as a non-bonded cutoff distance and distance-dependent dielectric. Then, 5.0 Å was set as an energy grid extending from the binding site [39]. The tested compounds were energy minimized inside the selected binding pocket. The editing and visualization of the generated binding poses were performed using Pymol software [40].

### 3.10. Molecular Dynamic Simulation

Molecular dynamic simulations (MDS) for ligand enzyme complexes were performed according to the previous protocol [41], using the Nanoscale Molecular Dynamics (NAMD) 2.6 software [42], applying the CHARMM27 force field [43]. Hydrogen atoms were added to the protein structures using the psfgen plugin included in the Visual Molecular Dynamic (VMD) 1.9 software [44]. Afterward, the whole system was solvated using TIP3P water particles and 0.15 M NaCl. The energy of the generated systems was first minimized and gradually heated to 300 K and equilibrated for 200/s. Subsequently, the MDS was continued for 20 ns, and the trajectory was stored every 0.1 ns and further analyzed with the VMD 1.9 software. The MDS output was sampled every 0.1 ns to evaluate the conformational changes of the entire system to analyze the root mean square deviation (RMSD) and root mean square fluctuation (RMSF). The topologies and parameters of the tested compounds were prepared using the VMD force field toolkit (ffTK) and the online software ligand reader and modeler (http://www.charmm-gui.org/?doc=input/ligandrm, accessed on 15 January 2021) [45]. MDS-derived binding free energies (Δ*G*) were calculated using the free energy perturbation (FEP) method through the web-based software Absolute Ligand Binder along with MDS using NAMD software [45,46]. Moreover, Δ*G* was calculated using another web-based software utilizing neural networking in its calculations, namely KDEEP (https://www.playmolecule.org/Kdeep/, accessed on 16 January 2021) [47].

### 3.11. Statistical Analysis

All in vitro experiments were performed in triplicate. Pooled data were presented as the mean ± standard error of the mean (SEM) of at least three independent experiments. The differences among various treatment groups were determined by ANOVA, followed by Dunnett’s test using PASW Statistics^®^ version 18 (Quarry Bay, Hong Kong). A difference of *p* < 0.05 was considered statistically significant and shown by a *symbol. The IC_50_ values were determined using a nonlinear regression curve fitting analysis using GraphPad Prism software version 6 (La Jolla, CA, USA).

## 4. Conclusions

Phytochemical investigation of the brown algae *S. cinereum* with the guidance of LC–HRESIMS dereplication afforded two new phenolic derivatives **12** and **13**, along with the known **19**, which exhibited moderate in vitro antiproliferative activity against HepG2, MCF-7, and Caco-2 cancer cell lines and considerable selective inhibition toward 5-LOX over 15-LOX. A series of in silico experiments (docking, MDS, and binding free energy calculations) were carried out to explore the mode of interaction of these compounds inside the active site of each enzyme. The present study shows the potential of marine natural products in providing unique metabolites with potent biological activities and highlighted the power of in silico investigations to facilitate drug discovery and development processes.

## Figures and Tables

**Table 1:** Distortionless enhancement by polarization transfer-Q (DEPT-Q) (400 MHz) and ^1^H (100 MHz) NMR data of compounds **12**, **13** in DMSO-*d_6_*; carbon multiplicities were determined by the DEPT-Q experiments.

Position	12		13	
	*^δ^*_C_	*^δ^*_H_ (*J* in Hz)	*^δ^*_C_	*^δ^*_H_ (*J* in Hz)
**1**	153.8, qC		153.8, qC	
**2**	123.1, CH	7.13, *d* (8.0)	123.1, CH	7.13, *d* (8.0)
**3**	123.6, CH	6.98, *d* (8.0)	123.6, CH	6.98, *d* (8.0)
**4**	134.5, qC		134.5, qC	
**5**	116.0, CH	6.68, s	140.6, qC	
**6**	140.6, qC		116.0, CH	6.68, s
**7**	29.8, CH_3_	1.34, *s*	31.9, CH_3_	1.23, *s*
**1′**	33.4, CH_2_	2.26, m	33.7, CH_2_	2.26, m
**2′**	20.3, CH_2_	2.03, overlapped	20.5, CH_2_	2.03, overlapped
**3′**	27.1, CH_2_	2.01, overlapped	27.1, CH_2_	2.01, overlapped
**4′**	127.9, CH	5.31–5.35, *m*	127.9, CH	5.31–5.35, *m*
**5′**	128.8, CH	5.31–5.35, *m*	128.8, CH	5.31–5.35, *m*
**6′**	25.5, CH_2_	2.78, overlapped	25.5, CH_2_	2.78, overlapped
**7′**	128.0, CH	5.31–5.35, *m*	128.1, CH	5.31–5.35, *m*
**8′**	128.3, CH	5.31–5.35, *m*	128.4, CH	5.31–5.35, *m*
**9** **′**	25.6, CH_2_	2.78, overlapped	25.6, CH_2_	2.78, overlapped
**10** **′**	128.2, CH	5.31–5.35, *m*	128.3, CH	5.31–5.35, *m*
**11** **′**	129.4, CH	5.31–5.35, *m*	129.4, CH	5.31–5.35, *m*
**12** **′**	24.9, CH_2_	1.52, overlapped	24.9, CH_2_	1.52, overlapped
**13** **′**	28.9, CH_2_	1.24, overlapped	28.9, CH_2_	1.24, overlapped
**14** **′**	22.4, CH_2_	1.25, overlapped	22.4, CH_2_	1.25, overlapped
**15** **′**	14.5, CH_3_	0.89, *t* (6.6)	14.3, CH_3_	0.85, *t* (6.6)

qC, quaternary, CH, methine, CH_2_, methylene, CH_3_, methyl carbons.

**Table 2:** In vitro antiproliferative activity of the isolated compounds, **12**–**21** expressed as IC_50_ ± (SSEM) µM.

IC_50_ (µM)			
Code	HepG2	MCF-7	Caco-2
**12**	14.5 ± 0.8 *	17.6 ± 0.9 *	18.2 ± 0.7 *
**13**	13.1 ± 1.1 *	12.7 ± 1.3 *	11.2 ± 0.6 *
**14**	>50	>50	>50
**15**	>50	>50	>50
**16**	>50	>50	>50
**17**	>50	>50	>50
**18**	>50	>50	>50
**19**	18.5 ± 1.4 *	21.6 ± 1.3*	15.7 ± 0.9 *
**20**	>50	>50	>50
**21**	>50	>50	>50
**Doxorubicin**	4.2 ± 0.3	3.8 ± 0.2	3.4 ± 0.1

The IC_50_ value of compounds against each cancer cell line, which was defined as the concentration (µM) that caused a 50% inhibition of cell growth in vitro, data were expressed as mean ± SEM (*n* = 3). One-way analysis of variance (ANOVA) followed by Dunnett’s test using PASW Statistics^®^ version 18 (Quarry Bay, Hong Kong) was applied. GraphPad Prism software version 6 (La Jolla, CA, USA) was used for statistical calculations. * Statistically significant at *p* < 0.05. Doxorubicin is a positive control.

**Table 3:** Docking scores, binding free energies, *K*_i_ and IC_50_ values of compounds **12**, **13**, and **19** together with the co-crystallized inhibitors NDGA and AA.

Compound	Δ*G*_Vina_ *		Δ*G*_FEP_ **		Δ*G_K_*_DEEP_ ***		*K*_i_^#^		IC_50_^#^	
	5-LOX	15-LOX	5-LOX	15-LOX	5-LOX	15-LOX	5-LOX	15-LOX	5-LOX	15-LOX
12	−9.3	−5.1	−8.1	−4.4	−7.7	−4.6	0.9 ± 0.1	17.4 ± 0.2	1.6 ± 0.3	25.3 ± 0.4
13	−8.9	−5.5	−8.0	−4.7	−7.5	−4.5	0.7 ± 0.2	14.3 ± 0.4	1.3 ± 0.1	23.6 ± 0.3
19	−9.1	−7.7	−7.9	−7.1	−7.6	−7.2	1.4 ± 0.2	4.2 ± 0.1	2.1 ± 0.4	6.7 ± 0.3
NDGA ^##^	−7.2	−6.9	−7.0	−6.5	−6.8	−6.5	6.9 ± 0.1	6.1 ± 0.2	8.8 ± 0.3	9.5 ± 0.5
AA ^##^	−7.6	−7.0	−6.2	−6.4	−7.1	−6.3	-	-	-	-

Lipoxygenase (LOX), nordihydroguaiaretic acid (NDGA), arachidonic acid (AA); * Vina docking scores calculated in kcal/mol; ** MDS-derived binding free energies calculated in kcal/mol by FEP method; *** neural networking-derived binding free energies calculated in kcal/mol by KDEEP software; ^#^ in vitro inhibition constant (*K*_i_) and inhibition concentration 50 (IC_50_) expressed as µM; ^##^ the reported co-crystalized ligands.

## Data Availability

Not applicable.

## Supplementary Materials

The following are available online. Table S1: Dereplicated metabolites from LC-HRESIMS analysis of Sarragassum cinnerum; Figure S1: LC-HRESIMS Chromatogram of the dereplicated metabolites of Sarragassum cinnerum (positive); Figure S2: LC-HRESIMS Chromatogram of the dereplicated metabolites of Sarragassum cinnerum (negative); Figure S3: ^1^H NMR spectrum of compound **12** measured in DMSO-d6 at 400 MHz; Figure S4: DEPT-Q NMR spectrum of compound **12** measured in DMSO-d6 at 100 MHz; Figure S5: HSQC spectrum of compound **12** measured in DMSO-d6; Figure S6: HMBC spectrum of compound **12** measured in DMSO-d6; Figure S7: HRESIMS spectrum of compound **12**; Figure S8: ^1^H NMR spectrum of compound **13** measured in DMSO-d6 at 400 MHz; Figure S9: DEPT-Q NMR spectrum of compound **13** measured in DMSO-d6 at 100 MHz; Figure S10: HSQC spectrum of compound **13** measured in DMSO-d6; Figure S11: HMBC spectrum of compound **13** measured in DMSO-d6; Figure S12: HRESIMS spectrum of compound **13**; Figure S13: ^1^H NMR spectrum of compound **14** measured in DMSO-d6 at 400 MHz; Figure S14: DEPT-Q NMR spectrum of compound **14** measured in DMSO-d6 at 100 MHz; Figure S15: ^1^H NMR spectrum of compound **15** measured in DMSO-d6 at 400 MHz; Figure S16: DEPT-Q NMR spectrum of compound **15** measured in DMSO-d6 at 100 MHz; Figure S17: ^1^H NMR spectrum of compound **16** measured in DMSO-d6 at 400 MHz; Figure S18: DEPT-Q NMR spectrum of compound **16** measured in DMSO-d6 at 100 MHz; Figure S19: ^1^H NMR spectrum of compound **17** measured in DMSO-d6 at 400 MHz; Figure S20: DEPT-Q NMR spectrum of compound **17** measured in DMSO-d6 at 100 MHz; Figure S21: ^1^H NMR spectrum of compound **18** measured in CD3OD-d4 at 400 MHz; Figure S22: DEPT-Q NMR spectrum of compound **18** measured in CD3OD-d6 at 100 MHz; Figure S23: ^1^H NMR spectrum of compound **19** measured in CD3OD-d4 at 400 MHz; Figure S24: DEPT-Q NMR spectrum of compound **19** measured in CD3OD-d6 at 100 MHz; Figure S25: ^1^H NMR spectrum of compound **20** measured in DMSO-d6 at 400 MHz; Figure S26: DEPT-Q NMR spectrum of compound **20** measured in DMSO-d6 at 100 MHz; Figure S27: ^1^H NMR spectrum of compound **21** measured in D2O at 400 MHz; Figure S28: DEPT-Q NMR spectrum of compound **21** measured in D2O at 100 MHz.

## References

[1] Guiry M. (2010). AlgaeBase. World-Wide Electronic Publication, National University of Ireland, Galway. https://ci.nii.ac.jp/naid/10028197296/.

[2] Yip Z.T., Quek R.Z., Huang D. (2020). Historical biogeography of the widespread *macroalga Sargassum* (Fucales, Phaeophyceae). J. Phycol..

[3] Yip Z.T., Quek R.Z., Low J.K., Wilson B., Bauman A.G., Chou L.M., Todd P.A., Huang D. (2018). Diversity and phylogeny of *Sargassum* (Fucales, Phaeophyceae) in Singapore. Phytotaxa.

[4] Rushdi M.I., Abdel-Rahman I.A., Saber H., Attia E.Z., Abdelraheem W.M., Madkour H.A., Hassan H.M., Elmaidomy A.H., Abdelmohsen U.R. (2020). Pharmacological and natural products diversity of the brown algae genus *Sargassum*. RSC Adv..

[5] Yende S.R., Harle U.N., Chaugule B.B. (2014). Therapeutic potential and health benefits of *Sargassum* species. Pharmacogn. Rev..

[6] Ma J., Zhang L., Zhang J., Liu M., Wei L., Shen T., Ma C., Wang Y., Chen Y., Zhu D. (2013). 15-Lipoxygenase-1/15-hydroxyeicosatetraenoic acid promotes hepatocellular cancer cells growth through protein kinase B and heat shock protein 90 complex activation. Int. J. Biochem. Cell Biol..

[7] Steele V.E., Holmes C.A., Hawk E.T., Kopelovich L., Lubet R.A., Crowell J.A., Sigman C.C., Kelloff G.J. (1999). Lipoxygenase inhibitors as potential cancer chemopreventives. Cancer Epidemiol. Prev. Biomark..

[8] Orafaie A., Matin M.M., Sadeghian H. (2018). The importance of 15-lipoxygenase inhibitors in cancer treatment. Cancer Metastasis Rev..

[9] Gilbert N.C., Gerstmeier J., Schexnaydre E.E., Börner F., Garscha U., Neau D.B., Werz O., Newcomer M.E. (2020). Structural and mechanistic insights into 5-lipoxygenase inhibition by natural products. Nat. Chem. Biol..

[10] Enomoto M., Kuwahara S. (2008). Enantioselective Synthesis and Stereochemical Revision of Communiols A–C, Antibacterial 2, 4-Disubstituted Tetrahydrofurans from the Coprophilous Fungus Podospora communis. Biosci. Biotechnol. Biochem..

[11] Takada N., Watanabe R., Suenaga K., Yamada K., Uemura D. (2001). Isolation and structures of hedaols A, B, and C, new bisnorditerpenes from a Japanese brown alga. J. Nat. Prod..

[12] Yoon W.-J., Heo S.-J., Han S.-C., Lee H.-J., Kang G.-J., Kang H.-K., Hyun J.-W., Koh Y.-S., Yoo E.-S. (2012). Anti-inflammatory effect of sargachromanol G isolated from *Sargassum siliquastrum* in RAW 264.7 cells. Arch. Pharmacal. Res..

[13] Gerasimenko N., Logvinov S. (2016). Seasonal composition of lipids, fatty acids pigments in the brown alga *Sargassum pallidum*: The potential for health. Open J. Mar. Sci..

[14] Iwashima M., Mori J., Ting X., Matsunaga T., Hayashi K., Shinoda D., Saito H., Sankawa U., Hayashi T. (2005). Antioxidant and antiviral activities of plastoquinones from the brown alga *Sargassum micracanthum*, and a new chromene derivative converted from the plastoquinones. Biol. Pharm. Bull..

[15] Tang H.-F., Yi Y.-H., Yao X.-S., Xu Q.-Z., Zhang S.-Y., Lin H.-W. (2002). Bioactive steroids from the brown alga *Sargassum carpophyllum*. J. Asian Nat. Prod. Res..

[16] Ayyad S.-E.N., Sowellim S.Z., El-Hosini M.S., Abo-Atia A. (2003). The structural determination of a new steroidal metabolite from the brown alga *Sargassum asperifolium*. Zeitschrift Naturforschung C.

[17] Jung M., Jang K.H., Kim B., Lee B.H., Choi B.W., Oh K.-B., Shin J. (2008). Meroditerpenoids from the brown alga *Sargassum siliquastrum*. J. Nat. Prod..

[18] Seo Y., Park K.E., Nam T.J. (2007). Isolation of a new chromene from the brown alga *Sargassum thunbergii*. Bull. Korean Chem. Soc..

[19] Cui Z., Li Y.-S., Liu H.-B., Yuan D., Lu B.-R. (2001). Sulfoglycolipid from the marine brown alga *Sargassum Hemiphyllum*. J. Asian Nat. Prod. Res..

[20] Ahamad P., Kunhi A., Divakar S. (2001). New metabolic pathway for *o*-cresol degradation by *Pseudomonas* sp. CP4 as evidenced by 1H NMR spectroscopic studies. World J. Microbiol. Biotechnol..

[21] Bogan L.E., Wolk S.K. (1992). Synthesis of and assignment of carbon-13 NMR resonances to *m*-cresol novolak dimers. Macromolecules.

[22] Tawfike A., Attia E.Z., Desoukey S.Y., Hajjar D., Makki A.A., Schupp P.J., Edrada-Ebel R., Abdelmohsen U.R. (2019). New bioactive metabolites from the elicited marine sponge-derived *bacterium Actinokineospora* spheciospongiae sp. nov. AMB Express.

[23] Wu W., Hasumi K., Peng H., Hu X., Wang X., Bao B. (2009). Fibrinolytic compounds isolated from a brown alga, *Sargassum fulvellum*. Mar. Drugs.

[24] Stappen I., Höfinghoff J., Buchbauer G., Wolschann P. (2010). Structure-activity relationships of *sandalwood odorants*: Synthesis of a new campholene derivative. Nat. Prod. Commun..

[25] Francesconi K.A., Edmonds J.S., Stick R.V., Skelton B.W., White A.H. (1991). Arsenic-containing ribosides from the brown alga *Sargassum lacerifolium*: X-ray molecular structure of 2-amino-3-[5′-deoxy-5′-(dimethylarsinoyl) ribosyloxy] propane-1-sulphonic acid. J. Chem. Soc. Perkin Trans. 1.

[26] Wang X., Shen Y., Wang S., Li S., Zhang W., Liu X., Lai L., Pei J., Li H. (2017). PharmMapper 2017 update: A web server for potential drug target identification with a comprehensive target pharmacophore database. Nucleic Acids Res..

[27] Xu X.-M., Deng J.-J., Yuan G.-J., Yang F., Guo H.-T., Xiang M., Ge W., Wu Y.-G. (2011). 5-Lipoxygenase contributes to the progression of hepatocellular carcinoma. Mol. Med. Rep..

[28] Hussey H., Tisdale M. (1996). Inhibition of tumour growth by lipoxygenase inhibitors. Br. J. Cancer.

[29] Avis I., Hong S.H., Martínez A., Moody T., Choi Y.H., Trepel J., Das R., Jett M., Mulshine J.L. (2001). Five-lipoxygenase inhibitors can mediate apoptosis in human breast cancer cell lines through complex eicosanoid interactions. FASEB J..

[30] Bishayee K., Khuda-Bukhsh A.R. (2013). 5-lipoxygenase antagonist therapy: A new approach towards targeted cancer chemotherapy. Acta Biochim. Biophys. Sin..

[31] Cer R.Z., Mudunuri U., Stephens R., Lebeda F.J. (2009). IC 50-to-K i: A web-based tool for converting IC 50 to K i values for inhibitors of enzyme activity and ligand binding. Nucleic Acids Res..

[32] Ashworth M.R.F., Stahl E. (2013). Thin-Layer Chromatography: A Laboratory Handbook.

[33] Abdelmohsen U.R., Cheng C., Viegelmann C., Zhang T., Grkovic T., Ahmed S., Quinn R.J., Hentschel U., Edrada-Ebel R. (2014). Dereplication strategies for targeted isolation of new antitrypanosomal actinosporins A and B from a marine sponge associated-*Actinokineospora* sp. EG49. Mar. Drugs.

[34] Skehan P., Storeng R., Scudiero D., Monks A., McMahon J., Vistica D., Warren J.T., Bokesch H., Kenney S., Boyd M.R. (1990). New colorimetric cytotoxicity assay for anticancer-drug screening. JNCI J. Natl. Cancer Inst..

[35] Vichai V., Kirtikara K. (2006). Sulforhodamine B colorimetric assay for cytotoxicity screening. Nat. Protoc..

[36] Yamamoto S. (1992). Mammalian lipoxygenases: Molecular structures and functions. Biochim. Biophys. Acta Lipids Lipid Metab..

[37] Mitra S., Bartlett S.G., Newcomer M.E. (2015). Identification of the substrate access portal of 5-lipoxygenase. Biochemistry.

[38] Trott O., Olson A.J. (2010). AutoDock Vina: Improving the speed and accuracy of docking with a new scoring function, efficient optimization, and multithreading. J. Comput. Chem..

[39] Sayed A.M., Alhadrami H.A., El-Gendy A.O., Shamikh Y.I., Belbahri L., Hassan H.M., Abdelmohsen U.R., Rateb M.E. (2020). Microbial natural products as potential inhibitors of SARS-CoV-2 main protease (Mpro). Microorganisms.

[40] Lill M.A., Danielson M.L. (2011). Computer-aided drug design platform using PyMOL. J. Comput. Aided Mol. Des..

[41] Alhadrami H.A., Hamed A.A., Hassan H.M., Belbahri L., Rateb M.E., Sayed A.M. (2020). Flavonoids as Potential anti-MRSA Agents through Modulation of PBP2a: A Computational and Experimental Study. Antibiotics.

[42] Phillips J.C., Braun R., Wang W., Gumbart J., Tajkhorshid E., Villa E., Chipot C., Skeel R.D., Kale L., Schulten K. (2005). Scalable molecular dynamics with NAMD. J. Comput. Chem..

[43] MacKerell A.D., Bashford D., Bellott M., Dunbrack R.L., Evanseck J.D., Field M.J., Fischer S., Gao J., Guo H., Ha S. (1998). All-atom empirical potential for molecular modeling and dynamics studies of proteins. J. Phys. Chem. B.

[44] Humphrey W., Dalke A., Schulten K. (1996). VMD: Visual molecular dynamics. J. Mol. Graph..

[45] Jo S., Kim T., Iyer V.G., Im W. (2008). CHARMM-GUI: A web-based graphical user interface for CHARMM. J. Comput. Chem..

[46] Jo S., Jiang W., Lee H.S., Roux B.t., Im W. (2013). CHARMM-GUI Ligand Binder for Absolute Binding Free Energy Calculations and Its Application. J. Chem. Inf. Model..

[47] Jiménez J., Skalic M., Martinez-Rosell G., De Fabritiis G. (2018). K deep: Protein–ligand absolute binding affinity prediction via 3d-convolutional neural networks. J. Chem. Inf. Model..

