# Supplementary material for: Potential Anticancer Lipoxygenase Inhibitors from the Red Sea-Derived Brown Algae Sargassum cinereum: An In-Silico-Supported In-Vitro Study
Source: Antibiotics (Basel). 2021 Apr 10;10(4):416. doi: 10.3390/antibiotics10040416 (PMC8069941; doi:10.3390/antibiotics10040416)
Supplement: Supplementary file 1 [file antibiotics-10-00416-s001.pdf]

SUPPLEMENTARY DATA

Potential Anticancer Lipoxygenase Inhibitors from the Red Sea-Derived Brown Algae *Sargassum cinereum*: An *In-Silico*-Supported *In-Vitro* Study

Sami I. Alzarea[1≠], Abeer H. Elmaidomy[2≠], Hani Saber[3], Arafa Musa[4,5], Mohammad M. Al-Sanea[6], Ehab M. Mostafa[4,5], Omnia Magdy Hendawy[1,7], Khayrya A. Youssif[8], Abdullah S. Alanazi[9], Metab Alharbi[10], Ahmed M. Sayed[11*], Usama Ramadan Abdelmohsen[12,13*]

[1]Department of Pharmacology, College of Pharmacy, Jouf University, Sakaka, Aljouf 72341, Saudi Arabia

[2]Department of Pharmacognosy, Faculty of Pharmacy, Beni-Suef University, Beni-Suef 62511, Egypt

[3]Department of Botany and Microbiology, Faculty of Science, South Valley University, 83523, Qena, Egypt

[4]Department of Pharmacognosy, College of Pharmacy, Jouf University 72341, Sakaka, Saudi Arabia

[5]Department of Pharmacognosy, Faculty of Pharmacy, Al-Azhar University, Cairo, 11371, Egypt

[6]Department of Pharmaceutical Chemistry, College of Pharmacy, Jouf University 72341, Sakaka, Saudi Arabia

[7]Clinical Pharmacology Department, Faculty of Medicine, Beni-Suef University, Egypt

[8]Department of Pharmacognosy, Faculty of Pharmacy, Modern University for Technology and Information, Cairo, Egypt

[9]Clinical Pharmacy Department, College of Pharmacy, Jouf University, Sakaka, Aljouf 72341, Saudi Arabia

[10]Department of Pharmacology and Toxicology, College of Pharmacy, King Saud University, P.O. Box 2457, Riyadh, 11451, Saudi Arabia.

[11]Department of Pharmacognosy, Faculty of Pharmacy, Nahda University, 62513, Beni-Suef, Egypt

[12]Department of Pharmacognosy, Faculty of Pharmacy, Minia University, Minia 61519, Egypt

[13]Department of Pharmacognosy, Faculty of Pharmacy, Deraya University, 7 Universities Zone, New Minia 61111, Egypt

*Corresponding authors: AMS: Department of Pharmacognosy, Faculty of Pharmacy, Nahda University, 62513, Beni-Suef, Egypt, ahmed.mohamed.sayed@nub.edu.eg; URA: Department of Pharmacognosy, Faculty of Pharmacy, Minia University, Minia 61519, Egypt, usama.ramadan@mu.edu.eg.

≠ Those authors have equally contributed.

List of Contents

Table S1. Dereplicated metabolites from LC-HRESIMS analysis of *Sarragassum cinnerum*

Figure S1. LC-HRESIMS Chromatogram of the dereplicated metabolites of *Sarragassum cinnerum* (positive)

Figure S2. LC-HRESIMS Chromatogram of the dereplicated metabolites of *Sarragassum cinnerum* (negative)

Figure S3. 1H NMR spectrum of compound **12** measured in DMSO-d_6 at 400 MHz

Figure S4. DEPT-Q NMR spectrum of compound **12** measured in DMSO-d_6 at 100 MHz

Figure S5. HSQC spectrum of compound **12** measured in DMSO-d_6

Figure S6. HMBC spectrum of compound **12** measured in DMSO-d_6

Figure S7. HRESIMS spectrum of compound **12**

Figure S8. 1H NMR spectrum of compound **13** measured in DMSO-d_6 at 400 MHz

Figure S9. DEPT-Q NMR spectrum of compound **13** measured in DMSO-d_6 at 100 MHz.

Figure S10. HSQC spectrum of compound **13** measured in DMSO-d_6

Figure S11. HMBC spectrum of compound **13** measured in DMSO-d_6

Figure S12. HRESIMS spectrum of compound **13**

Figure S13. 1H NMR spectrum of compound **14** measured in DMSO-d_6 at 400 MHz

Figure S14. DEPT-Q NMR spectrum of compound **14** measured in DMSO-d_6 at 100 MHz.

Figure S15. 1H NMR spectrum of compound **15** measured in DMSO-d_6 at 400 MHz

Figure S16. DEPT-Q NMR spectrum of compound **15** measured in DMSO-d_6 at 100 MHz

Figure S17. 1H NMR spectrum of compound **16** measured in DMSO-d_6 at 400 MHz

Figure S18. DEPT-Q NMR spectrum of compound **16** measured in DMSO-d_6 at 100 MHz

Figure S19. 1H NMR spectrum of compound **17** measured in DMSO-d_6 at 400 MHz

Figure S20. DEPT-Q NMR spectrum of compound **17** measured in DMSO-d_6 at 100 MHz

Figure S21. 1H NMR spectrum of compound **18** measured in CD_3OD-d_4 at 400 MHz

Figure S22. DEPT-Q NMR spectrum of compound **18** measured in CD_3OD-d_6 at 100 MHz

Figure S23. 1H NMR spectrum of compound **19** measured in CD_3OD-d_4 at 400 MHz

Figure S24. DEPT-Q NMR spectrum of compound **19** measured in CD_3OD-d_6 at 100 MHz

Figure S25. 1H NMR spectrum of compound **20** measured in DMSO-d_6 at 400 MHz

Figure S26. DEPT-Q NMR spectrum of compound **20** measured in DMSO-d_6 at 100 MHz

Figure S27. 1H NMR spectrum of compound **21** measured in D_2O at 400 MHz

Figure S28. DEPT-Q NMR spectrum of compound **21** measured in D_2O at 100 MHz

Table S1. Dereplicated metabolites using the Dictionary of Natural Products database from LC-HRESIMS analysis of *Sarragassum cinnerum*

No.	Metabolites name	Original source	MF	RT(min.)	*m/z*	MS^2 Fragmentation	Ionization Mode
**1**	(5R,7S,8S)-Communiol A	*Sargassum spp.*	$C_{11}H_{20}O_4$	3.76288	215.1283	119.1183, 153.1283, 179.1075	$[M-H]^-$
**2**	Hedaol A	*Sargassum spp.*	$C_{18}H_{30}O_2$	6.7354	277.2162	237.1864, 219.1752	$[M-H]^-$
**3**	Arachidonic acid	*Sargassum pallidum*	$C_{20}H_{34}O_2$	6.6166	307.2624	289.2528, 271.2424, 245.2266	$[M+H]^+$
**4**	Sargachromanol A	*Sargassum siliquastrum*	$C_{22}H_{30}O_3$	5.3686	343.2276	325.2158, 285.1853, 273.1850	$[M+H]^+$
**5**	2-Geranylgeranyl-6-methylbenzoquinone	*Sargassum micracanthum.*	$C_{27}H_{38}O_2$	4.4710	395.2950	259.2425, 257.1534	$[M+H]^+$
**6**	24-Ethylcholesta-4,24(28)-dien-3,6-dione	*Sargassum carpophyllum*	$C_{29}H_{44}O_2$	6.5544	425.3420	313.2157, 285.1854	$[M+H]^+$
**7**	Saringosterone	*Sargassum asperfolium*	$C_{29}H_{46}O_2$	6.6324	427.3576	411.3262, 391.3363, 177.1277	$[M+H]^+$
**8**	Nahocol A	*Sargassum siliquastrum*	$C_{29}H_{42}O_6$	6.6773	487.3060	459.3109, 373.2375	$[M+H]^+$
**9**	24xi-Hydroperoxy-24-vinylcholesterol	*Sargassum carpophyllum*	$C_{29}H_{48}O_3$	6.5615	445.3682	373.2215	$[M+H]^+$
**10**	Sargathunbergol A	*Sargassum thunbergii*	$C_{27}H_{38}O_6$	5.7502	459.2749	285.1855, 257.1534	$[M+H]^+$
**11**	1-O-(11-Hexadecenoyl)-3-0-(6'-sulfo-a-D-quinovopyranosyl) glycerol	*Sargassum hemiphyllum*	$C_{25}H_{46}O_{11}S$	4.4836	553.2681	-	$[M-H]^-$

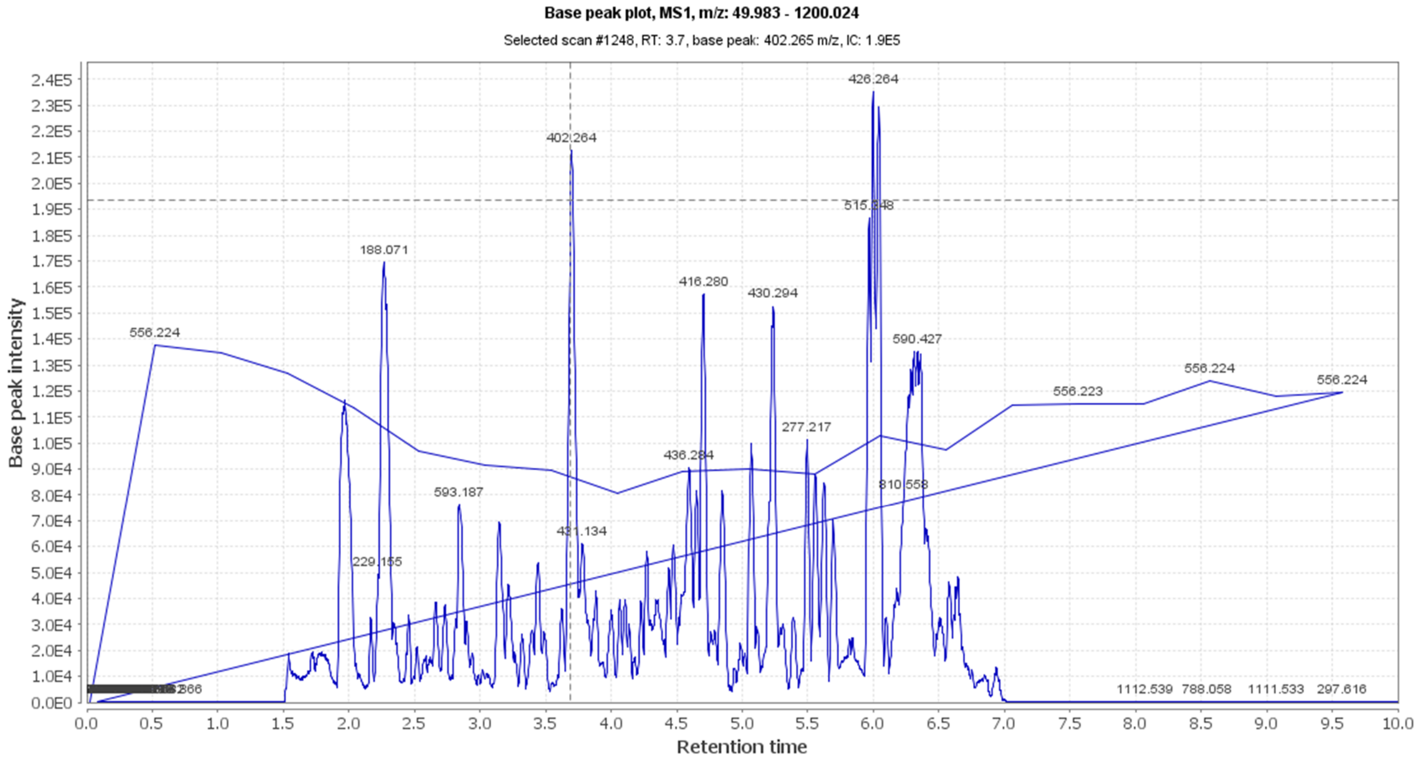

Figure S1. LC-HRESIMS Chromatogram of the dereplicated metabolites of *Sarragassum cinnerum* (positive)

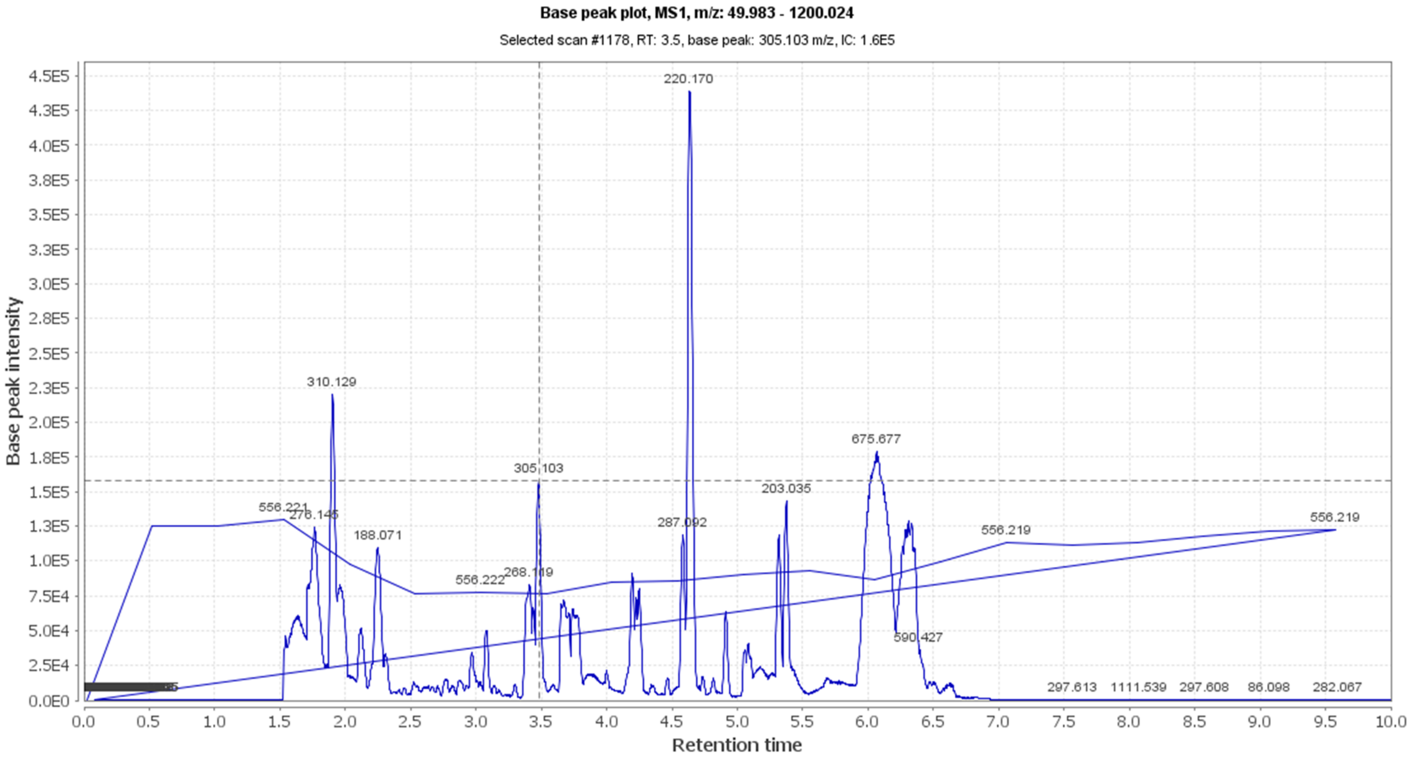

Figure S2. LC-HRESIMS Chromatogram of the dereplicated metabolites of *Sarragassum cinnerum* (negaive)

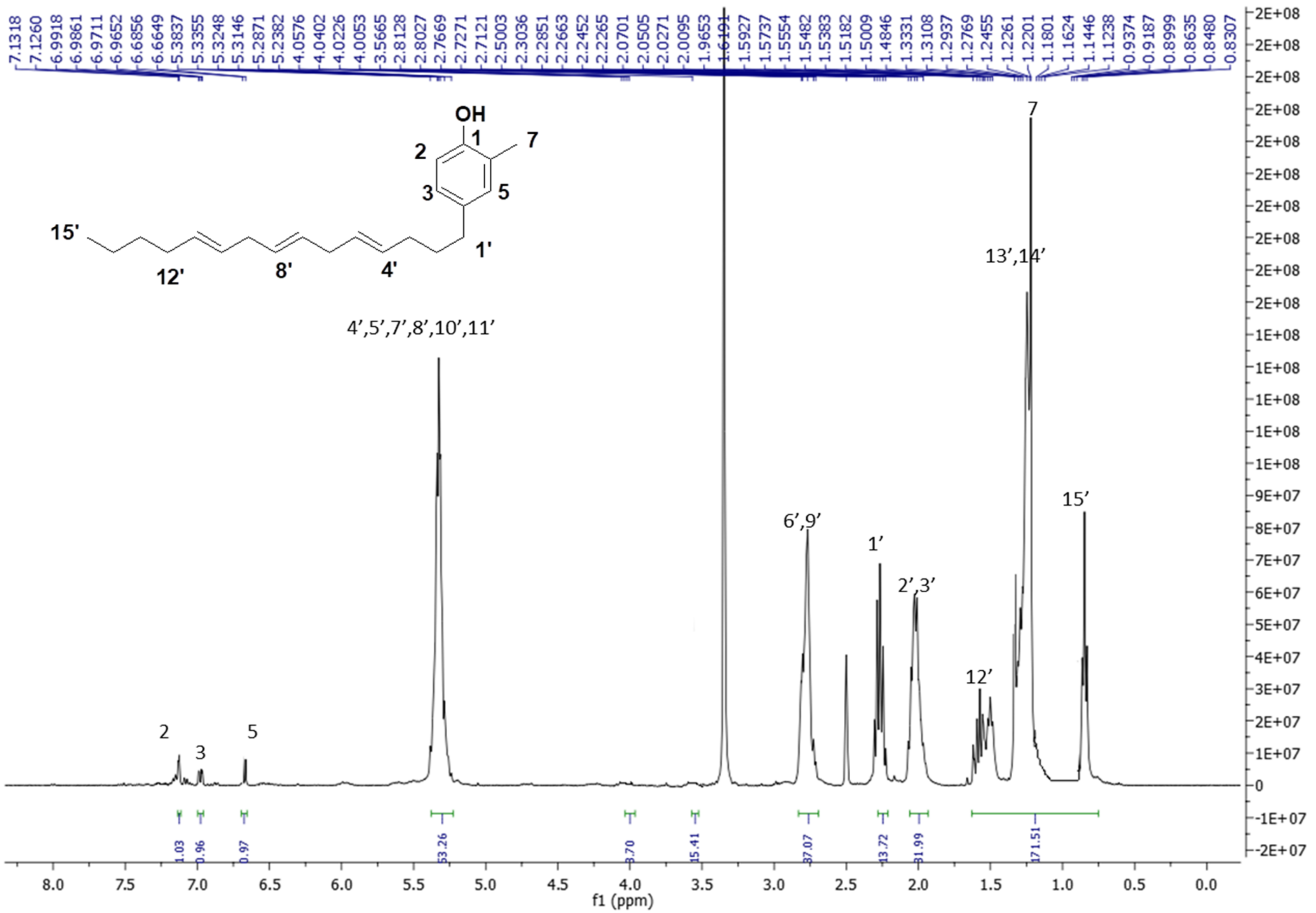

Figure S3. 1H NMR spectrum of compound **12** measured in DMSO-d_6 at 400 MHz

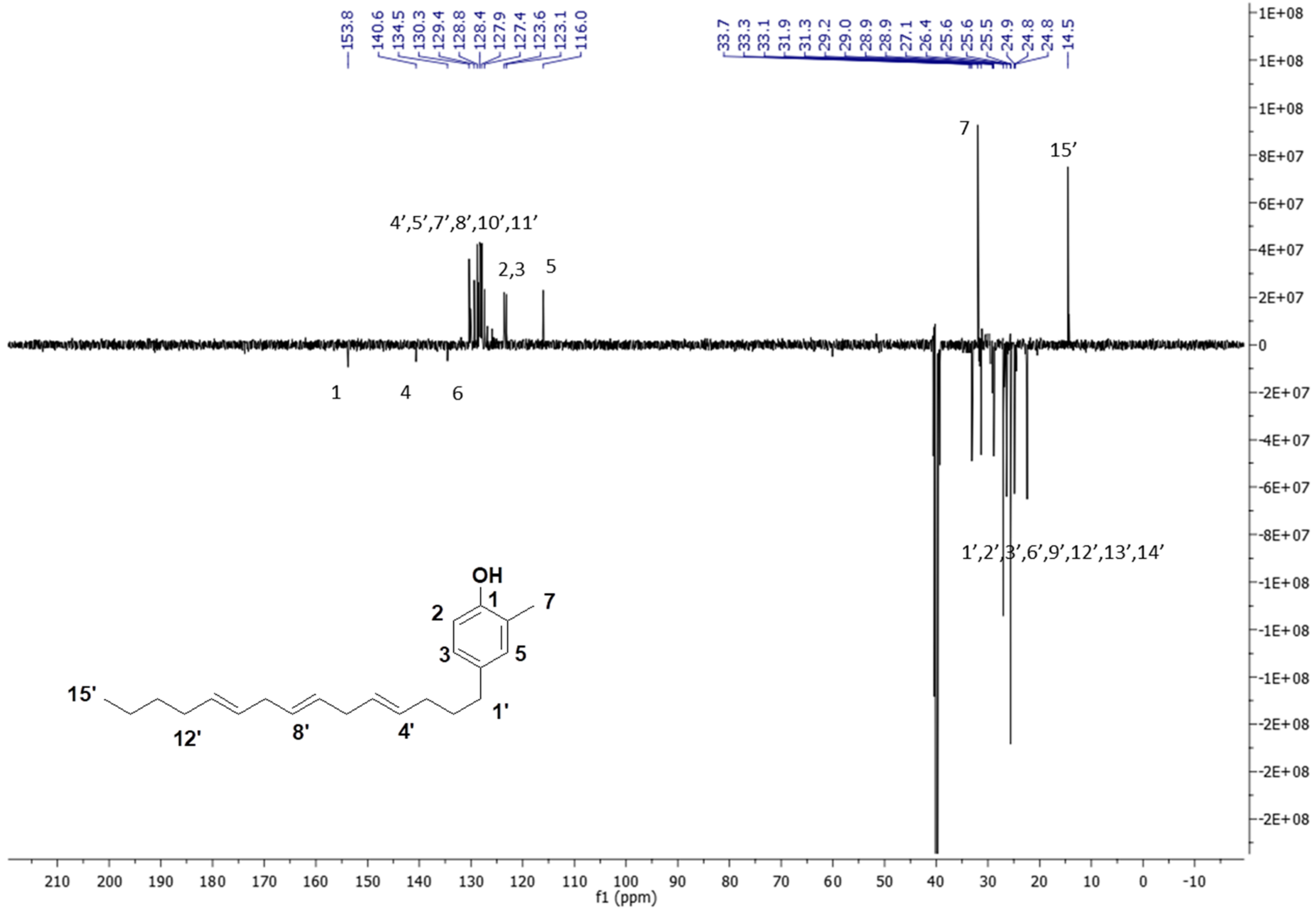

Figure S4. DEPT-Q NMR spectrum of compound **12** measured in DMSO-d_6 at 100 MHz.

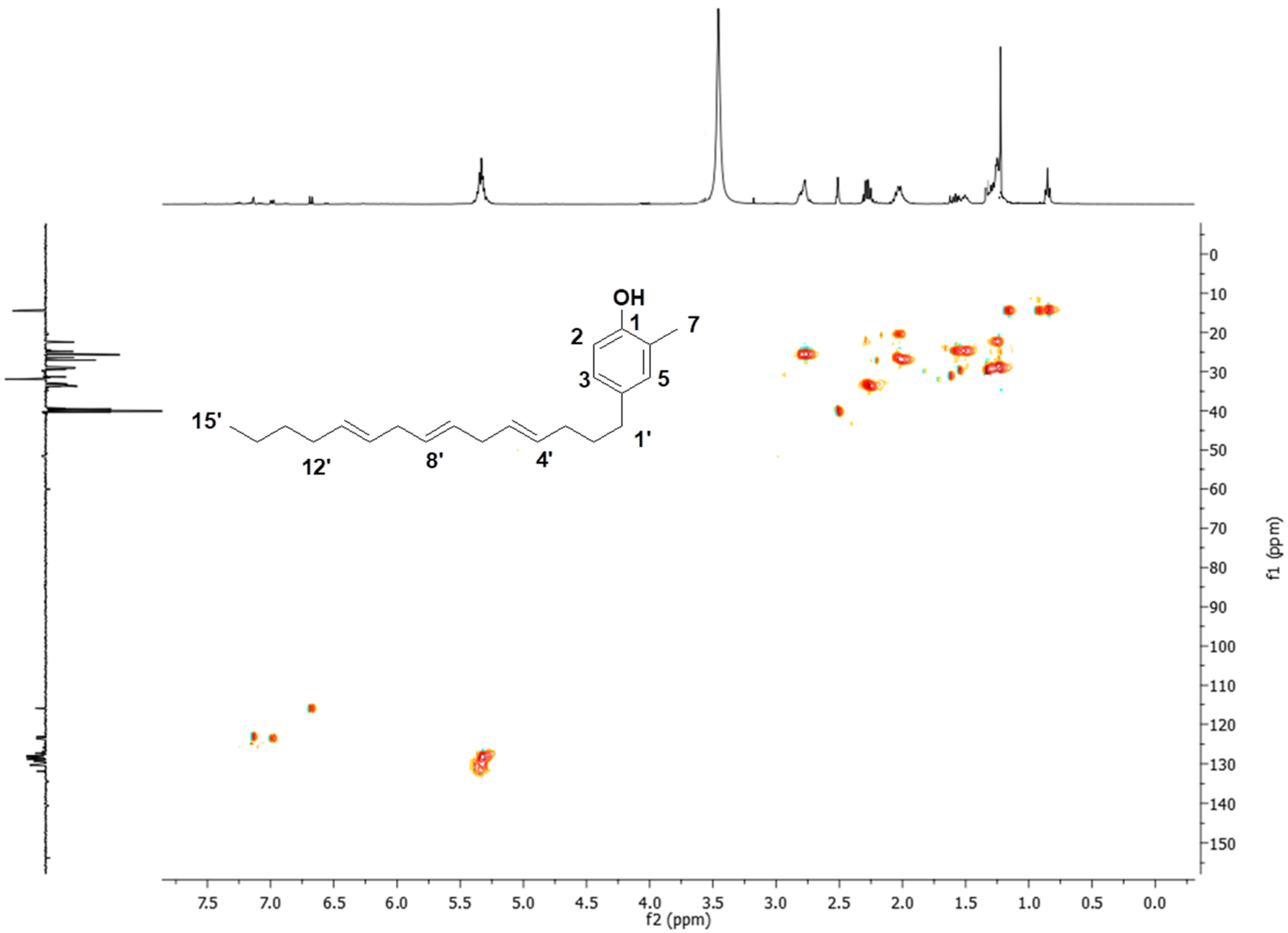

Figure S5. HSQC spectrum of compound **12** measured in DMSO-d_6

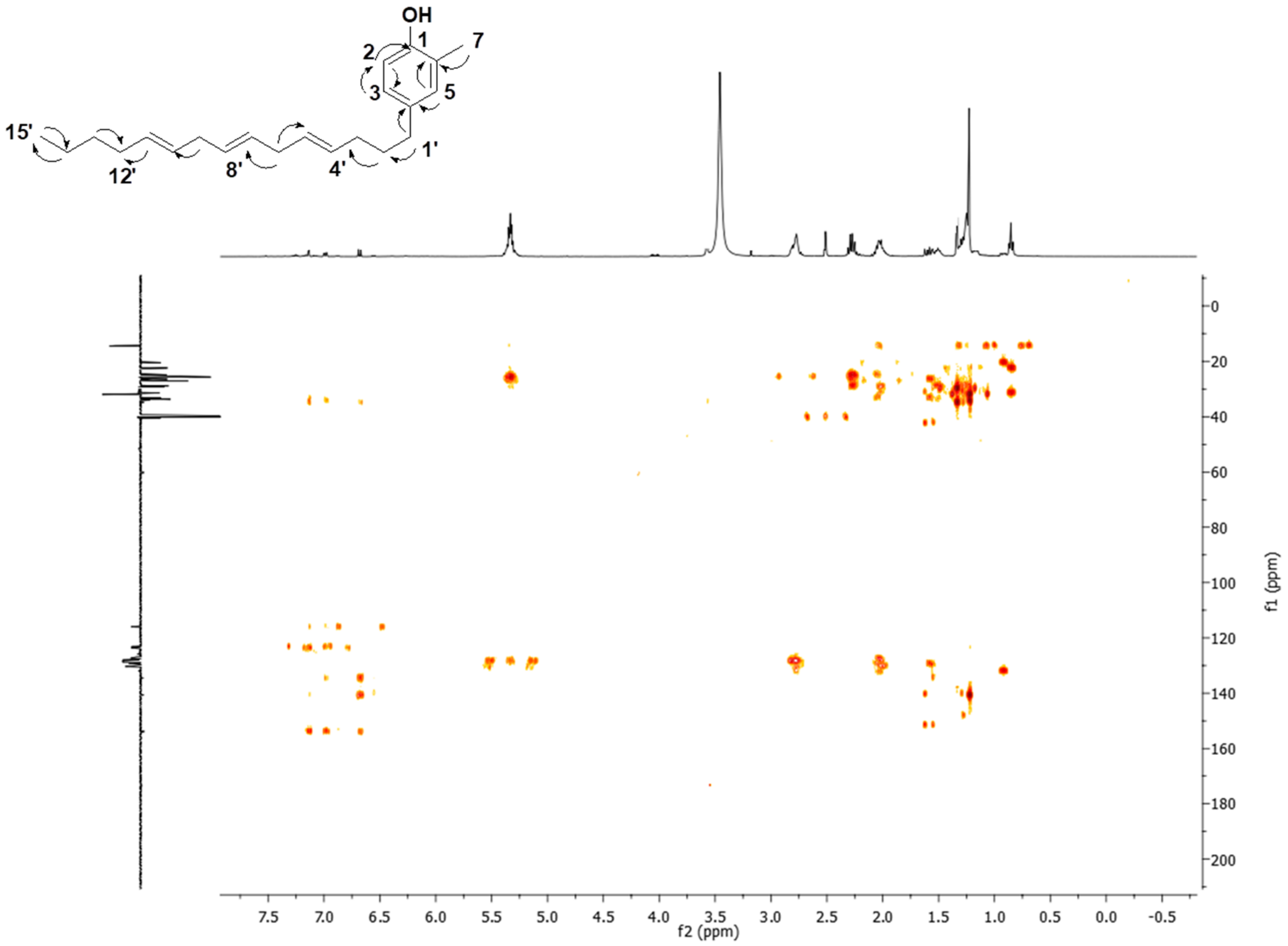

Figure S6. HMBC spectrum of compound **12** measured in DMSO-d_6

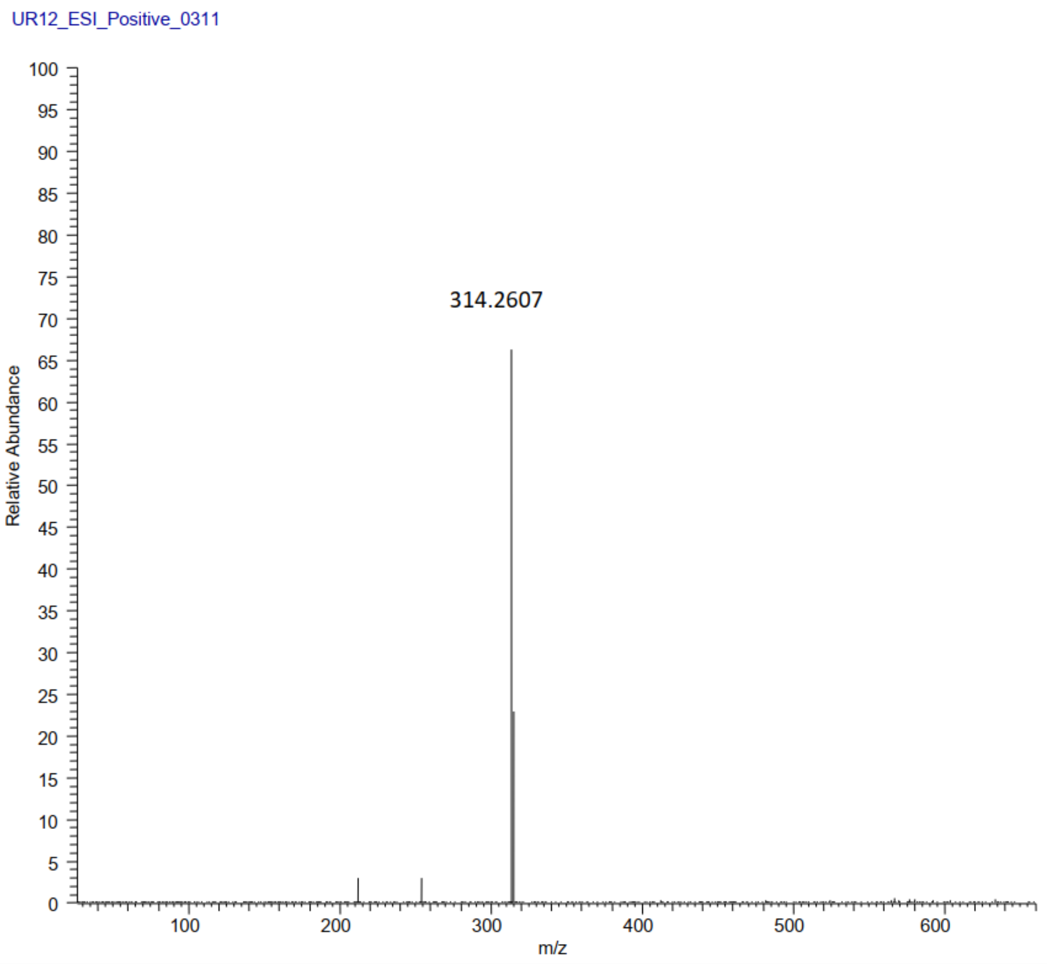

Figure S7. HRESIMS spectrum of compound **12**

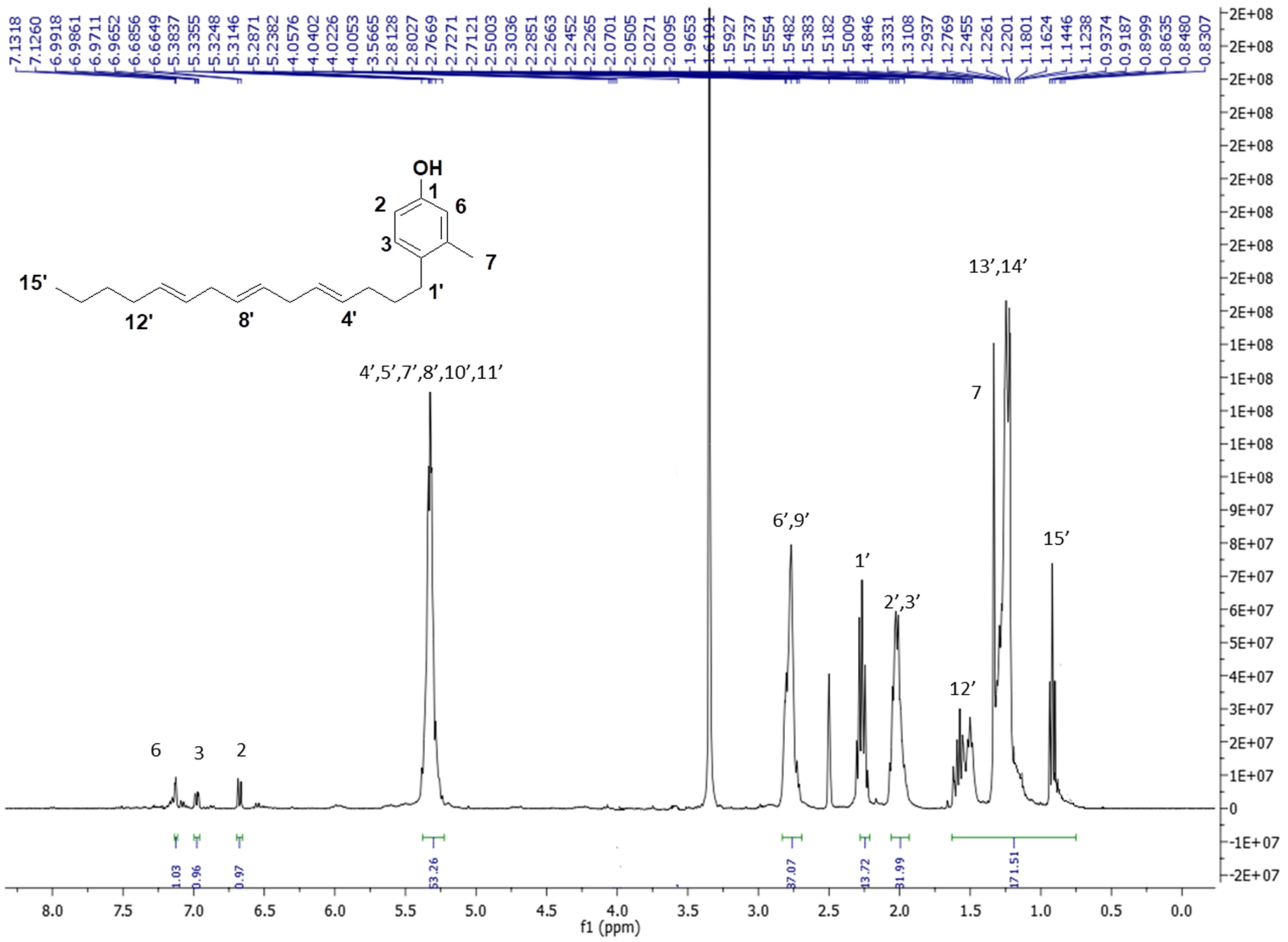

Figure S8. 1H NMR spectrum of compound **13** measured in DMSO-d_6 at 400 MHz

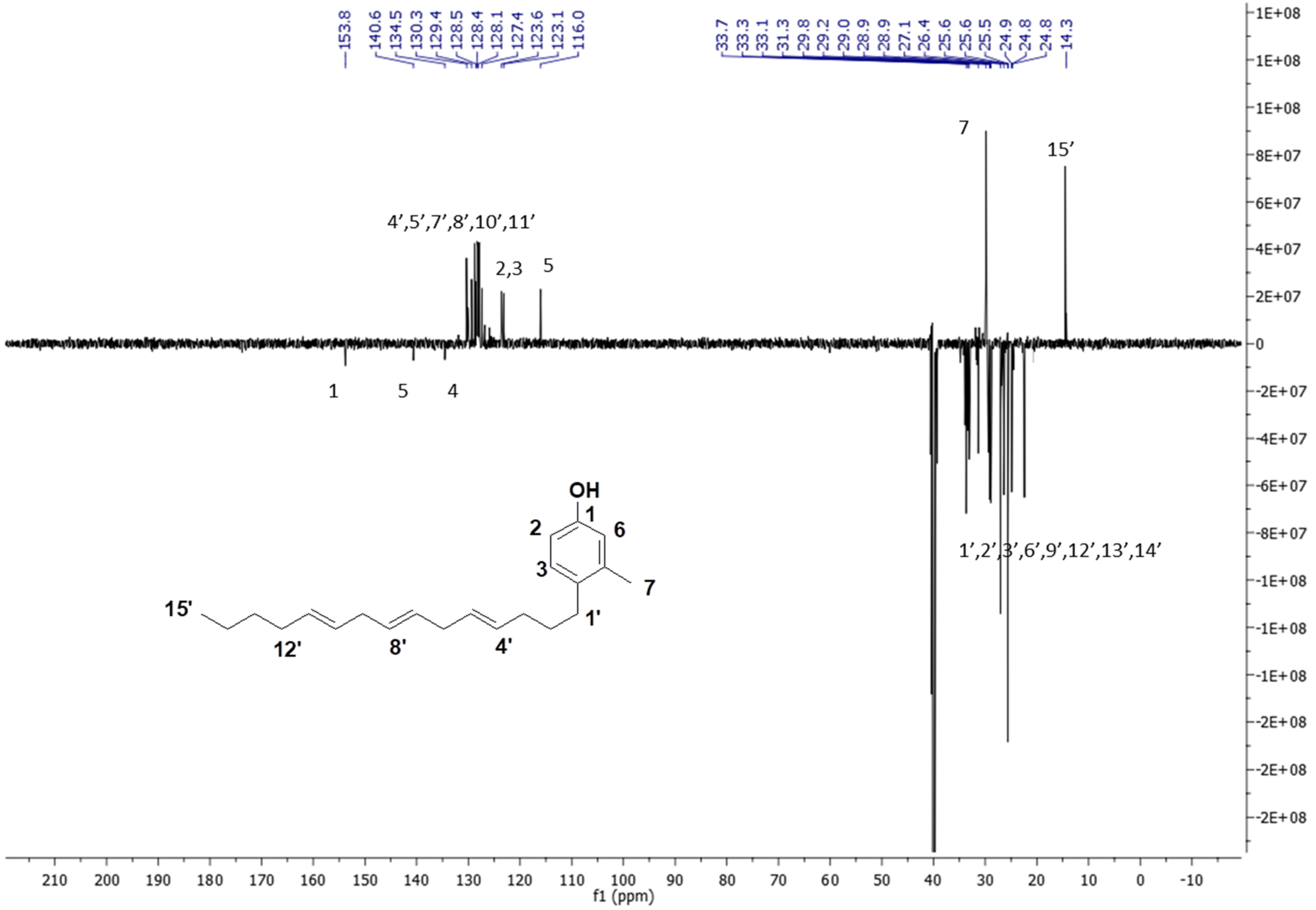

Figure S9. DEPT-Q NMR spectrum of compound **13** measured in DMSO-d_6 at 100 MHz.

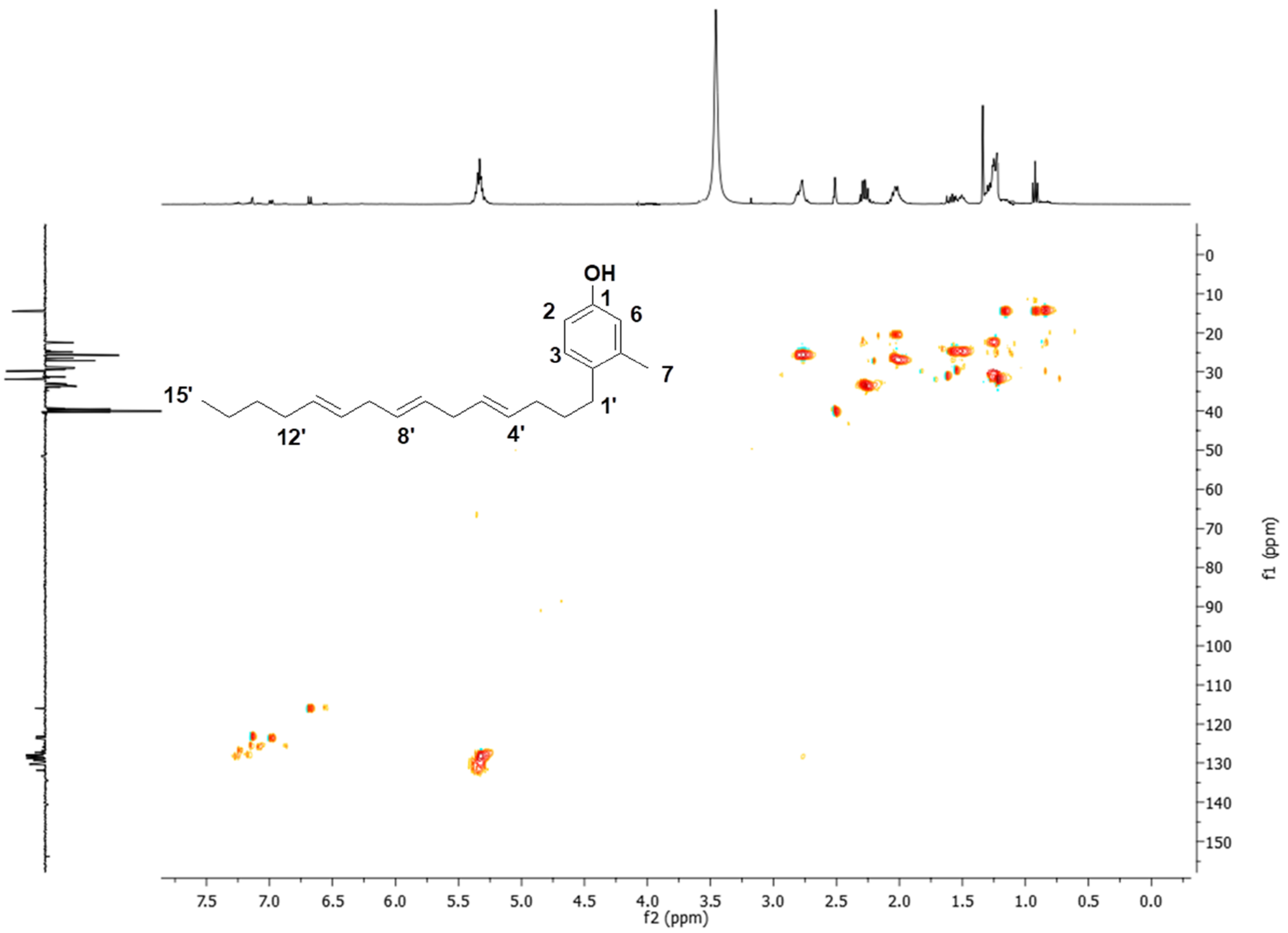

Figure S10. HSQC spectrum of compound **13** measured in DMSO-d_6

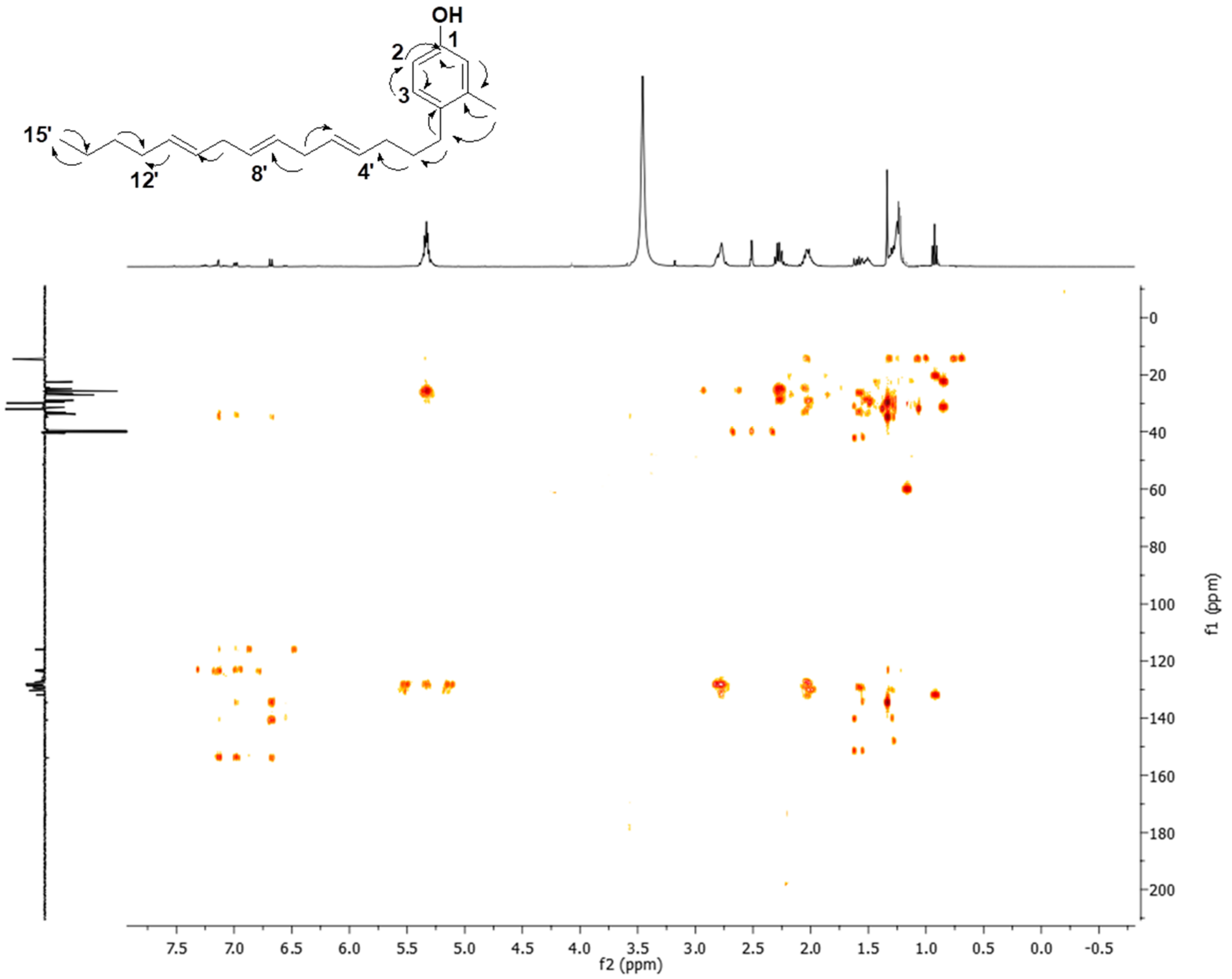

Figure S11. HMBC spectrum of compound **13** measured in DMSO-d_6

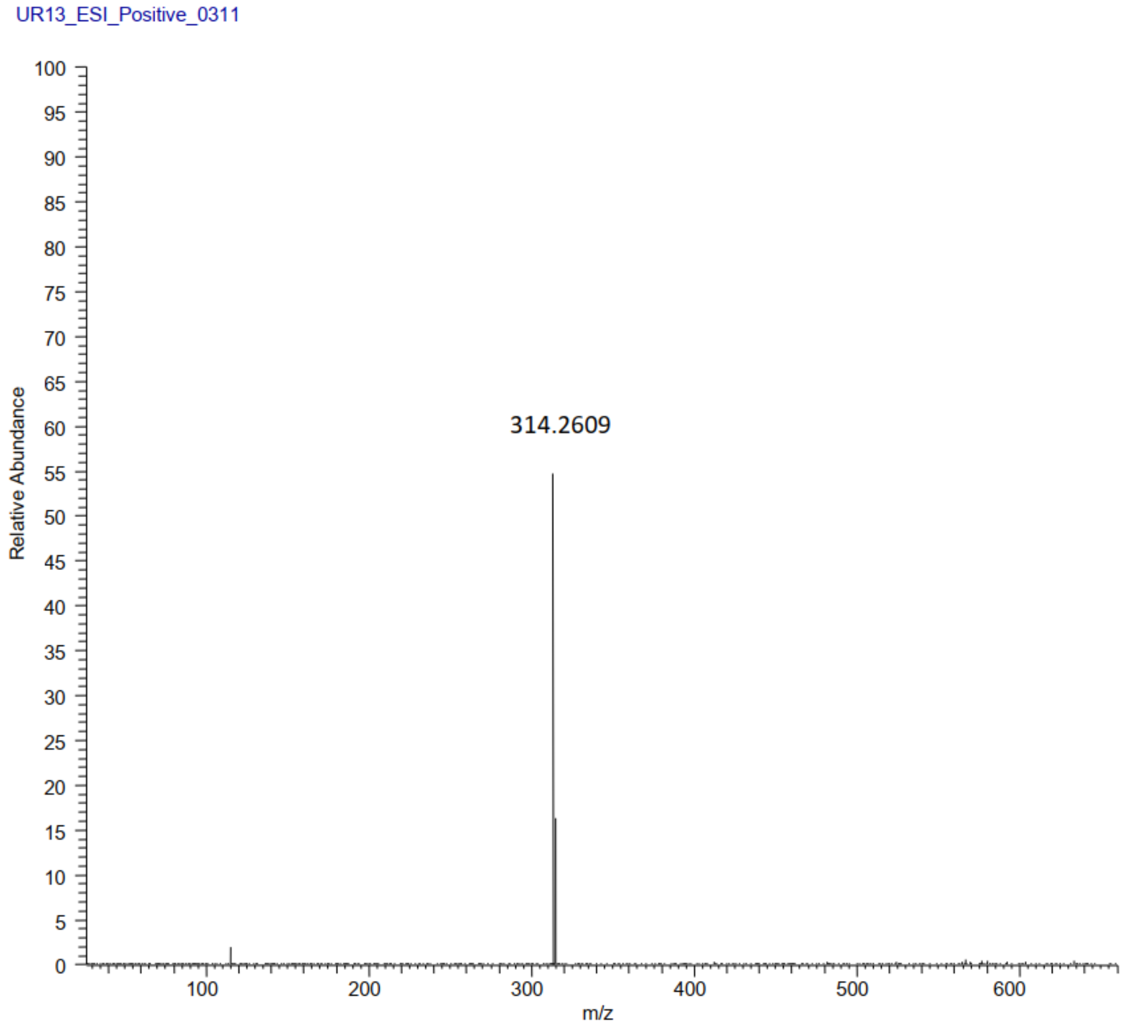

Figure S12. HRESIMS spectrum of compound **13**

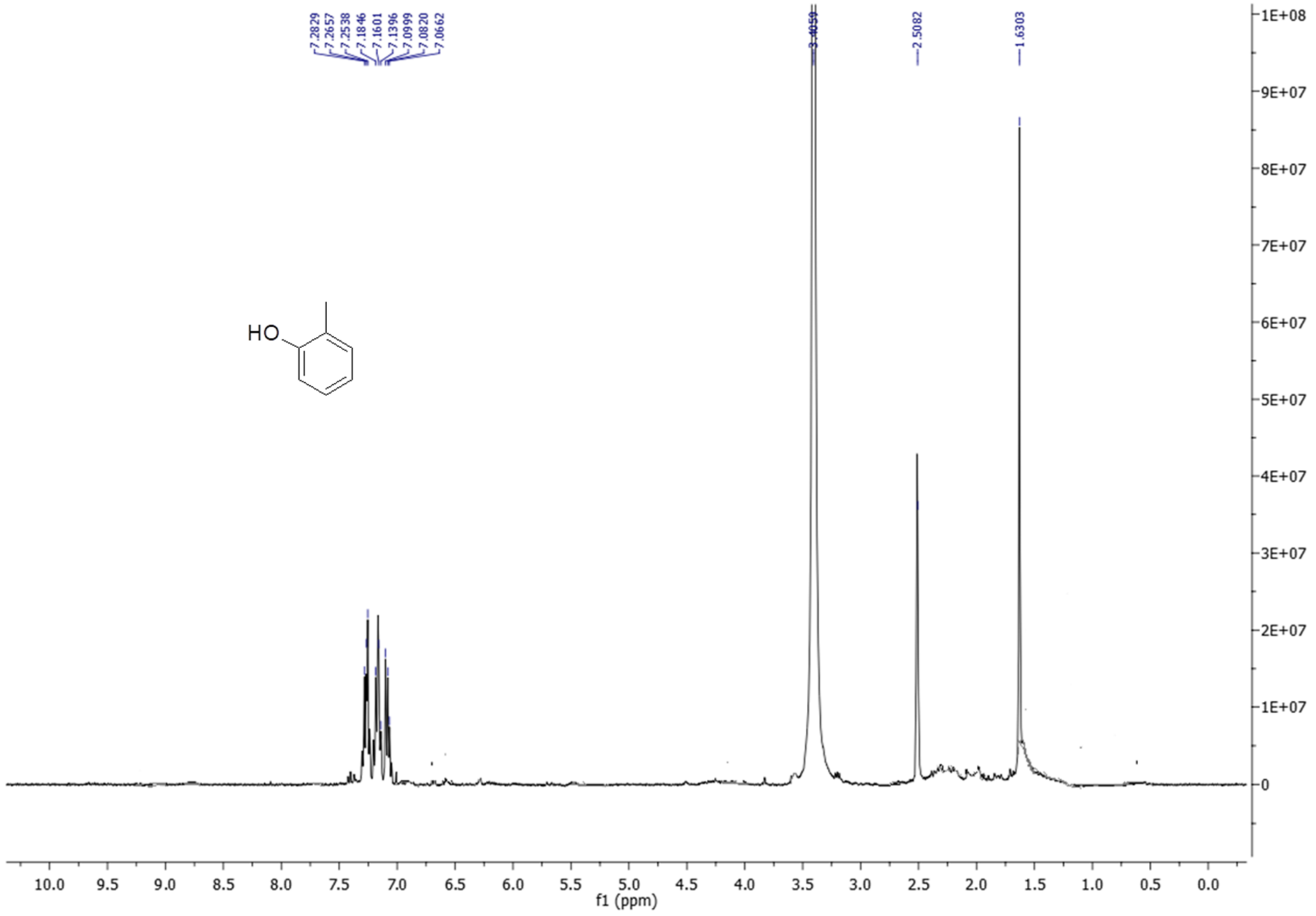

Figure S13. 1H NMR spectrum of compound **14** measured in DMSO-d_6 at 400 MHz

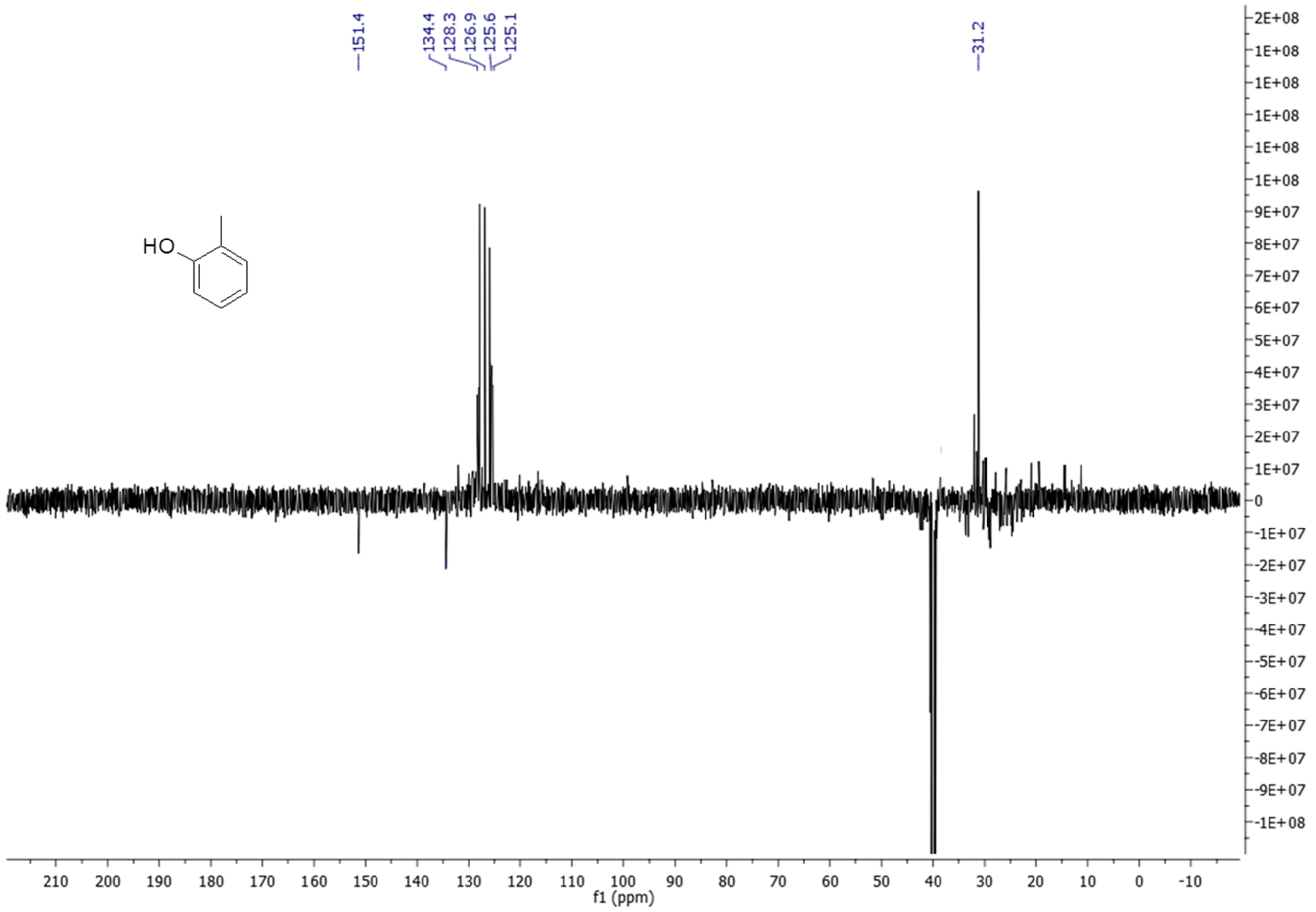

Figure S14. DEPT-Q NMR spectrum of compound **14** measured in DMSO-d_6 at 100 MHz.

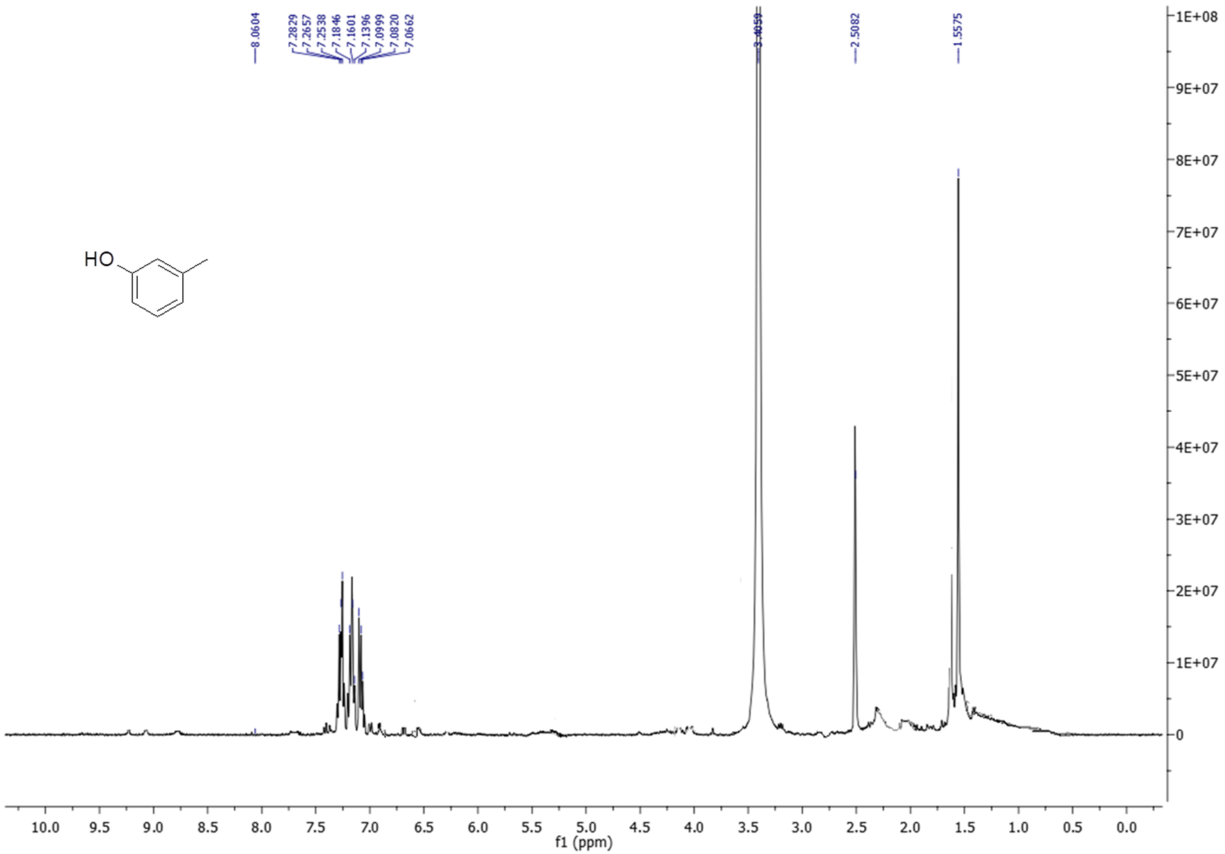

Figure S15. 1H NMR spectrum of compound **15** measured in DMSO-d_6 at 400 MHz

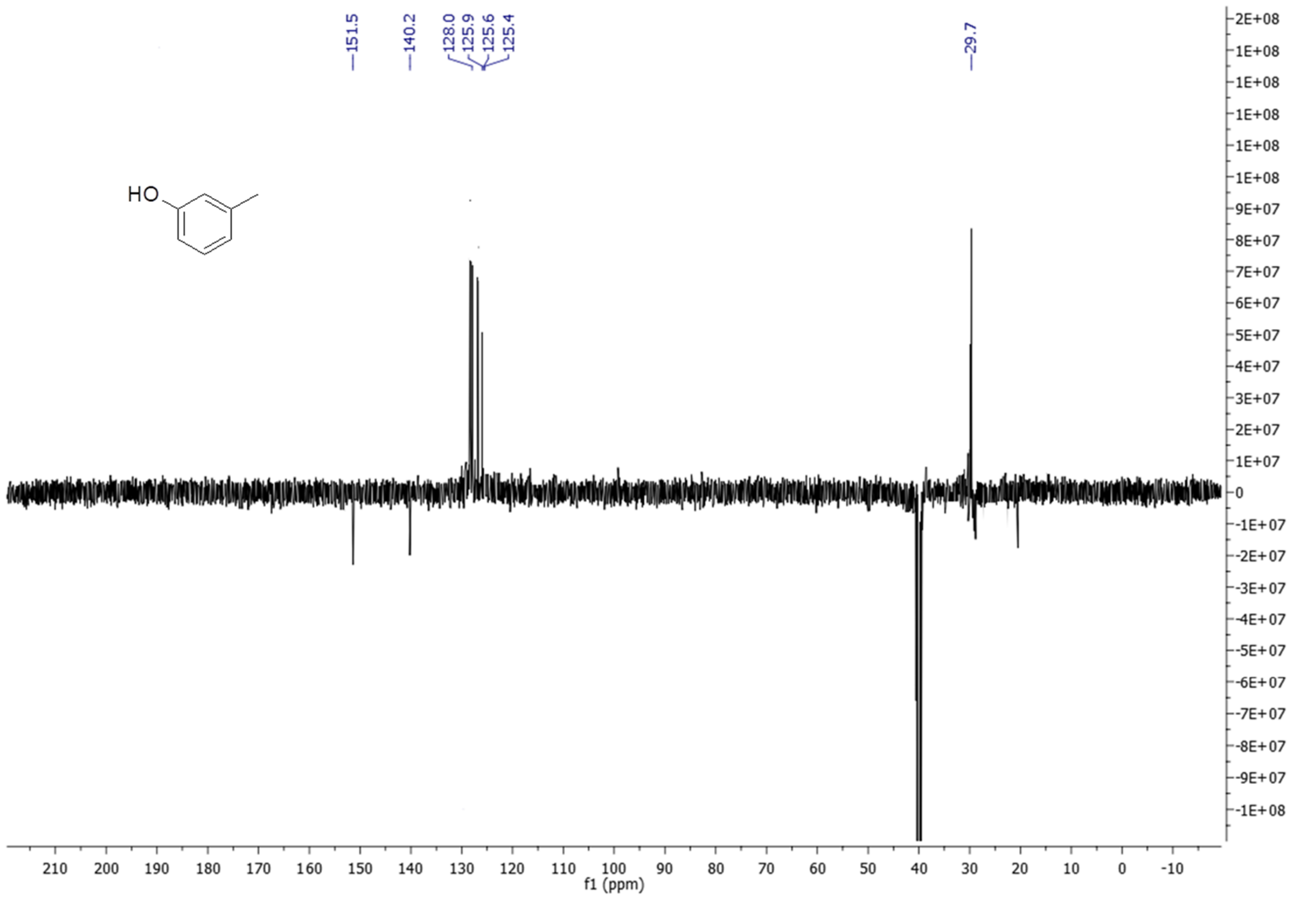

Figure S16. DEPT-Q NMR spectrum of compound **15** measured in DMSO-d_6 at 100 MHz

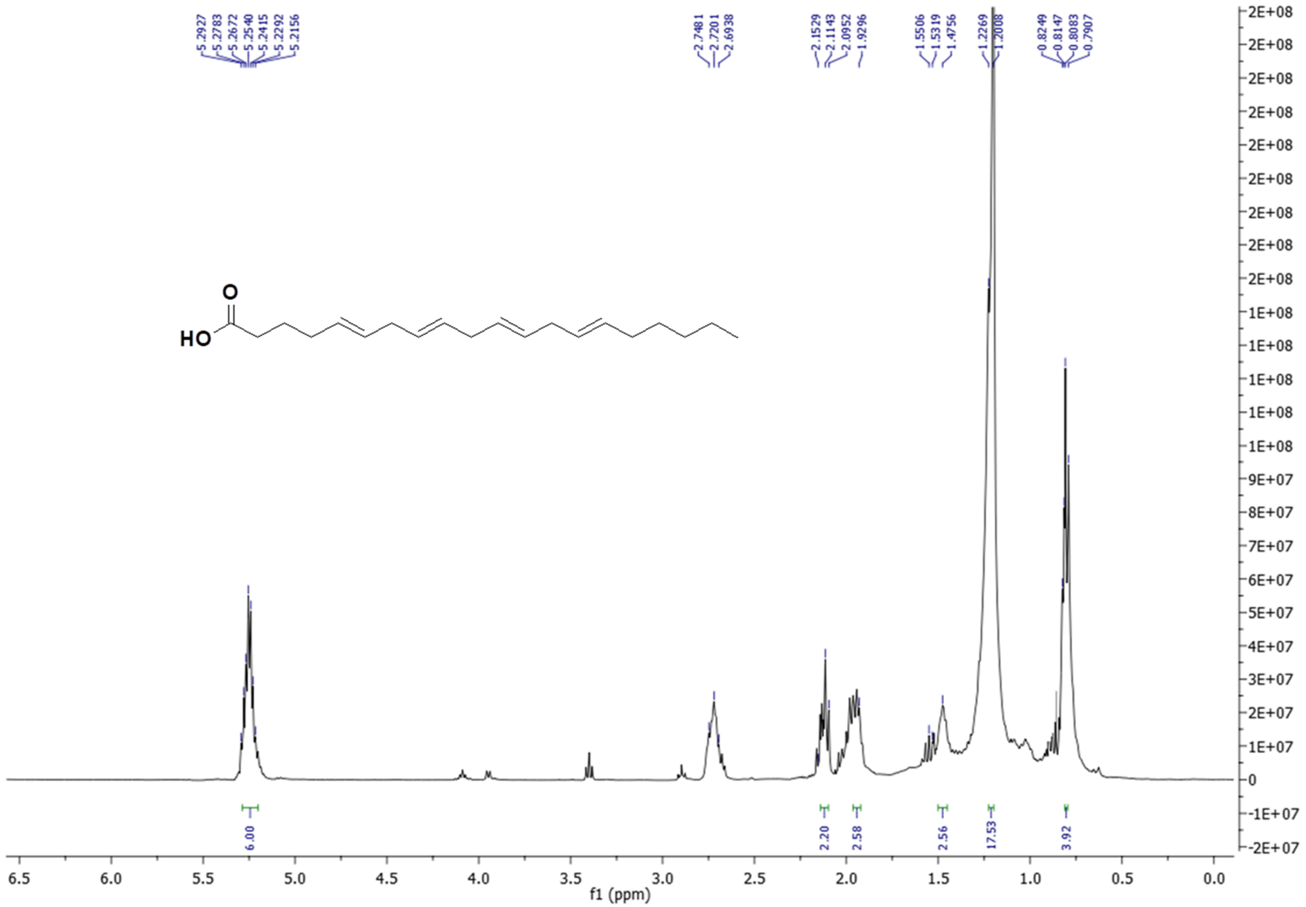

Figure S17. 1H NMR spectrum of compound **16** measured in DMSO-d_6 at 400 MHz

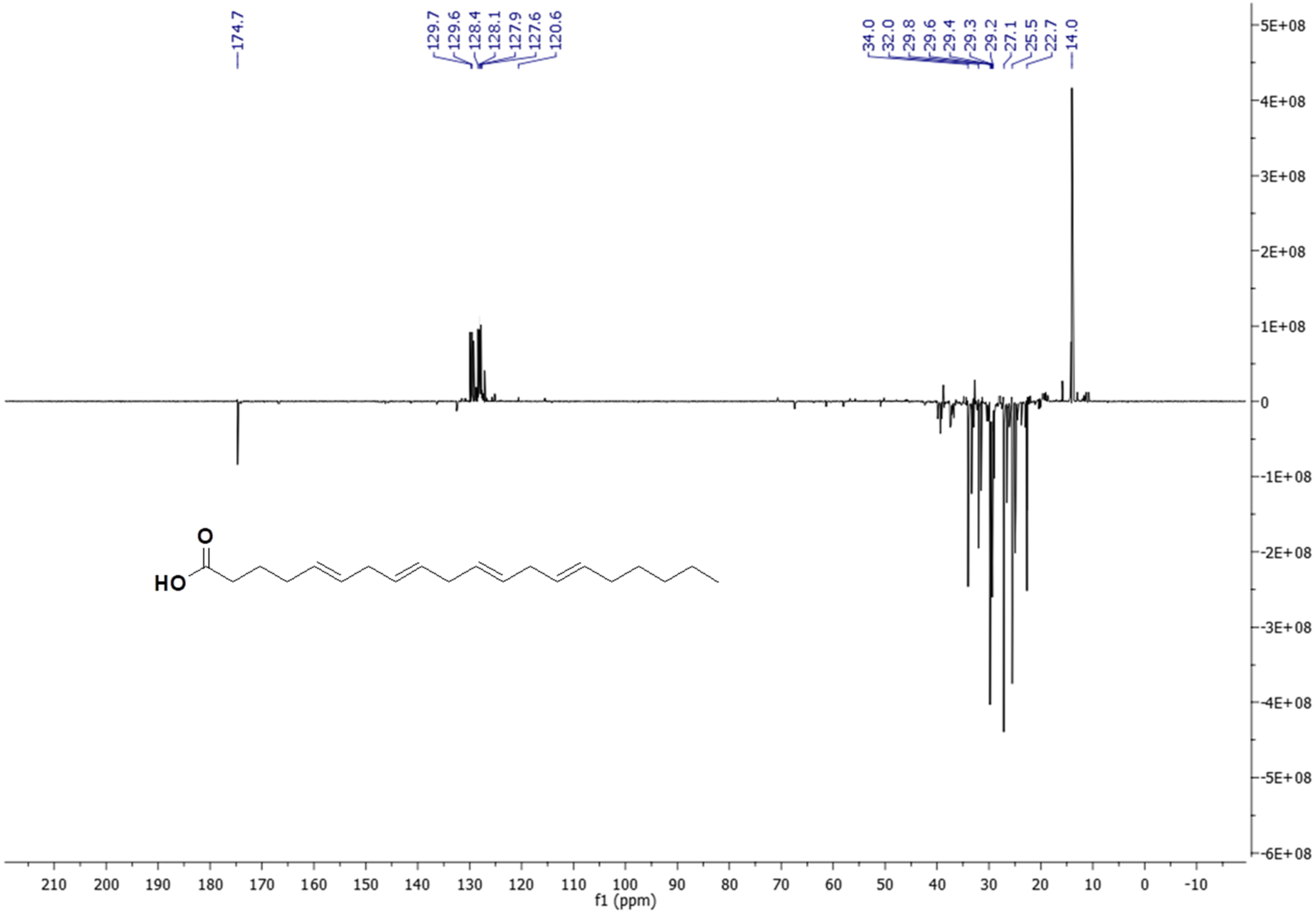

Figure S18. DEPT-Q NMR spectrum of compound **16** measured in DMSO-d_6 at 100 MHz

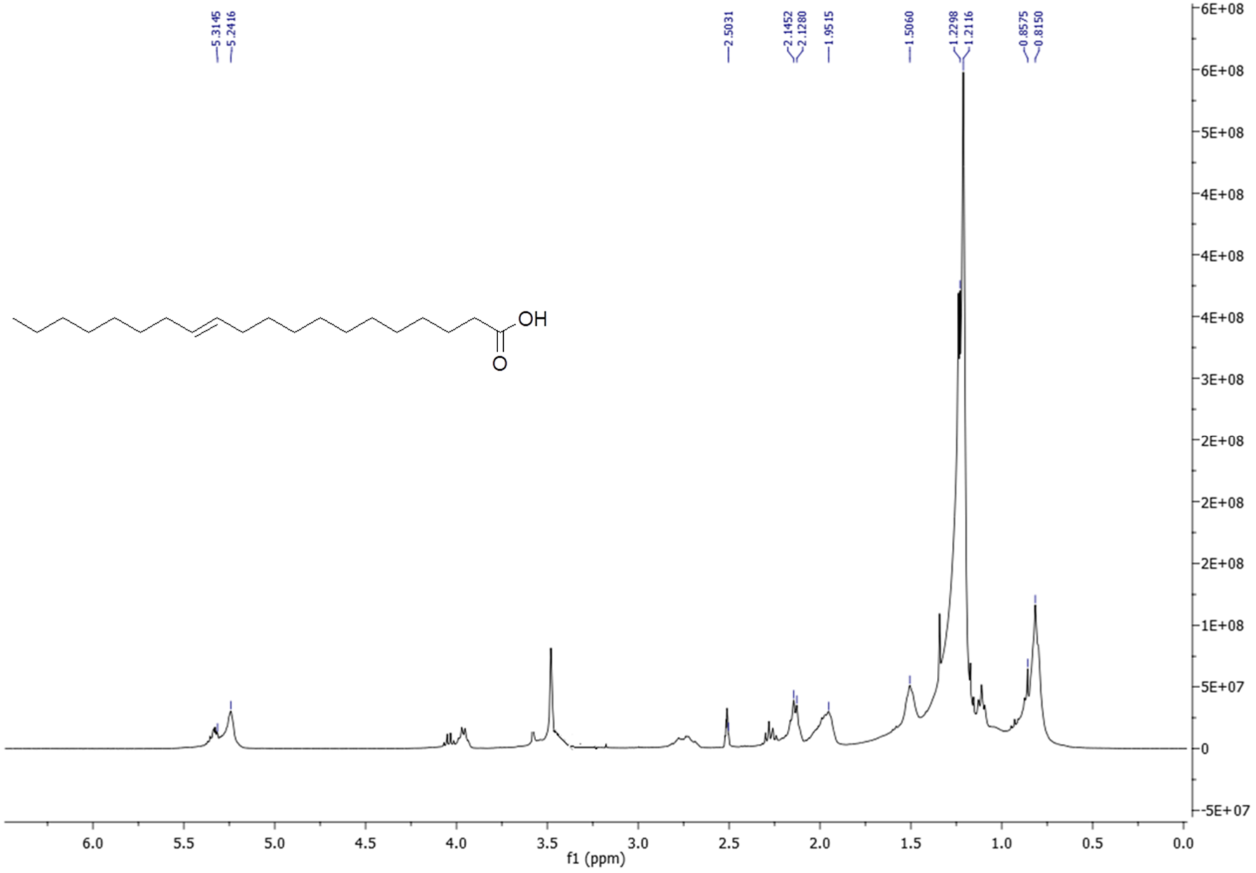

Figure S19. 1H NMR spectrum of compound **17** measured in DMSO-d_6 at 400 MHz

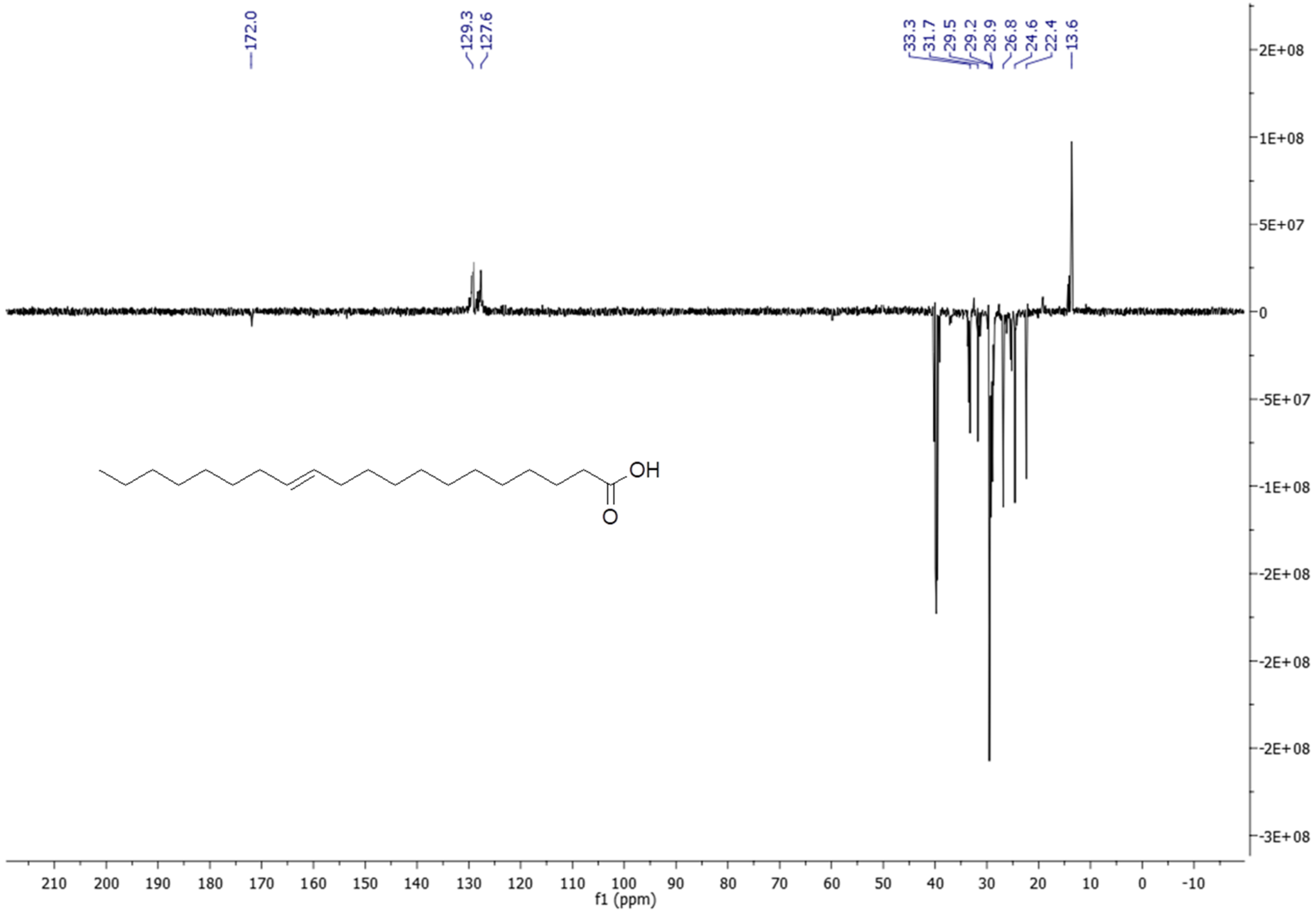

Figure S20. DEPT-Q NMR spectrum of compound **17** measured in DMSO-d_6 at 100 MHz

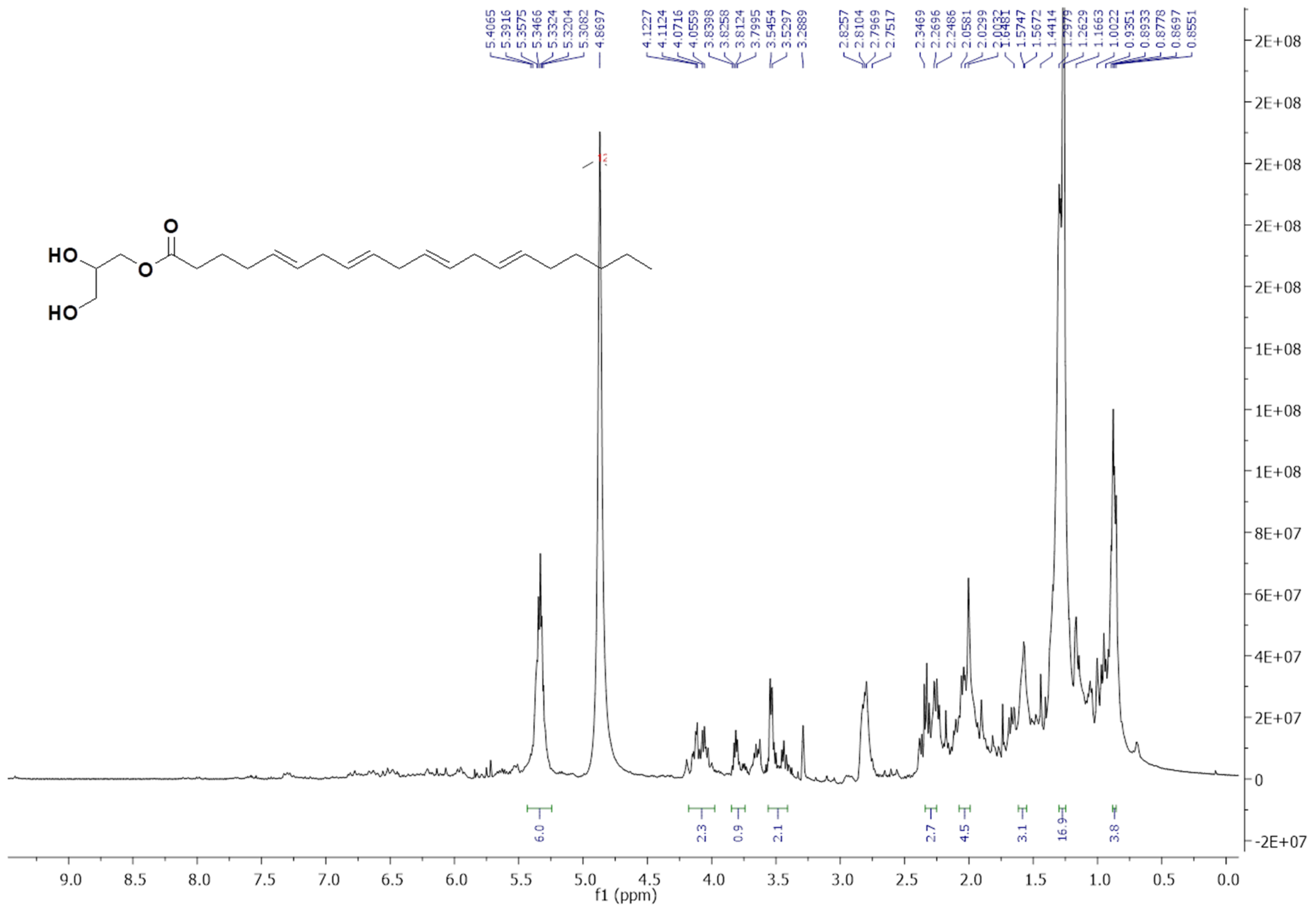

Figure S21. 1H NMR spectrum of compound **18** measured in CD_3OD-d_4 at 400 MHz

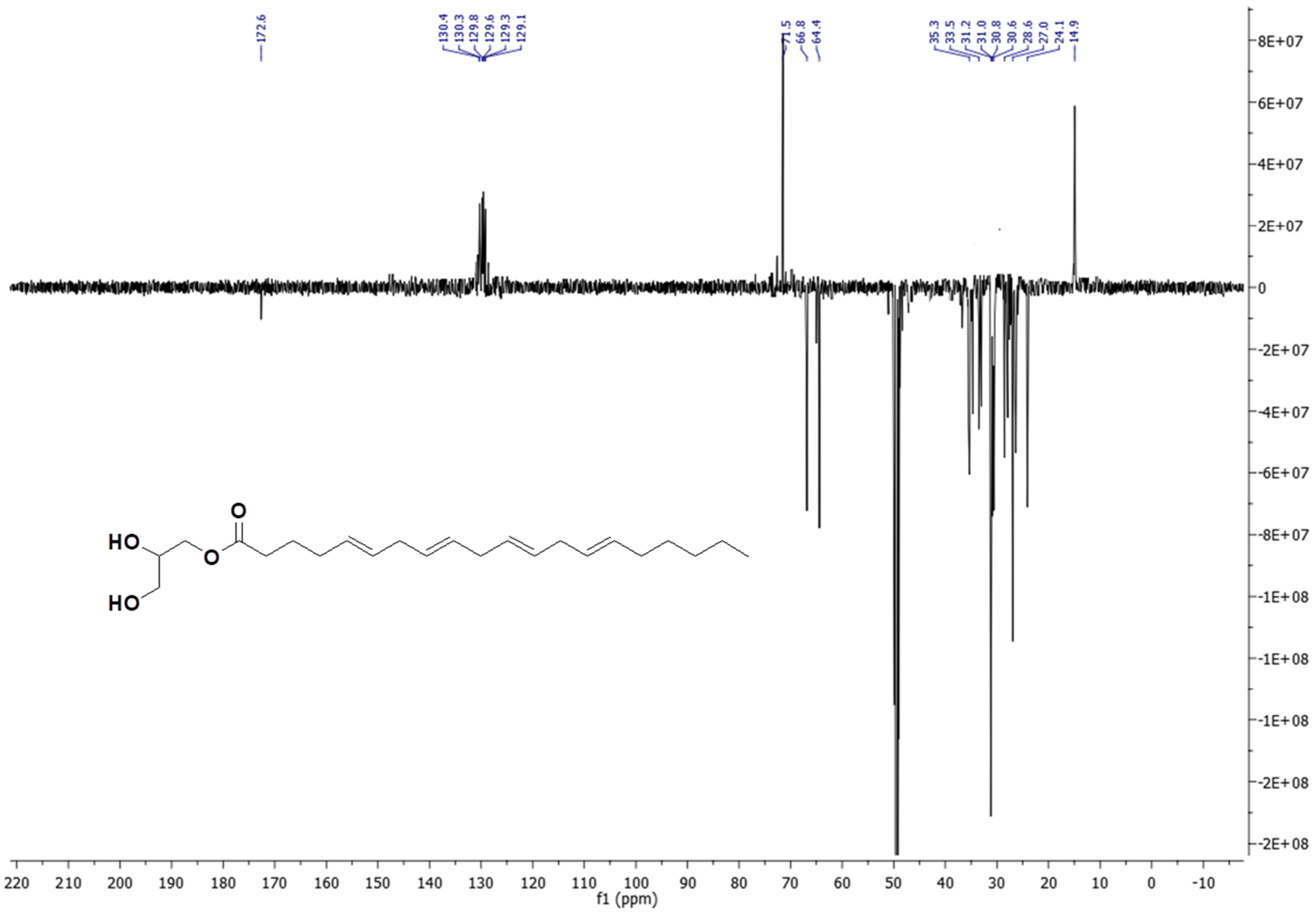

Figure S22. DEPT-Q NMR spectrum of compound **18** measured in CD_3OD-d_6 at 100 MHz

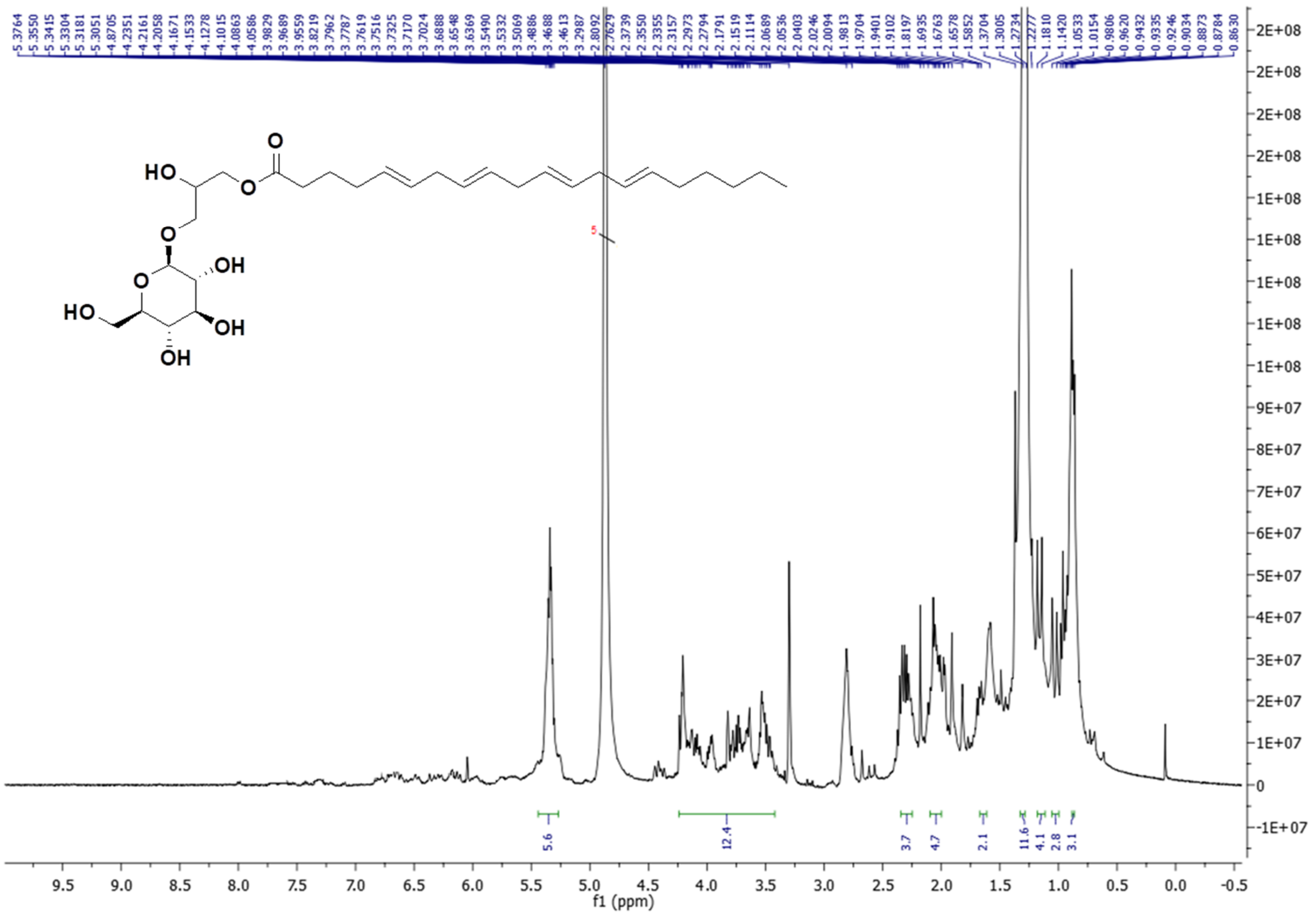

Figure S23. 1H NMR spectrum of compound **19** measured in CD_3OD-d_4 at 400 MHz

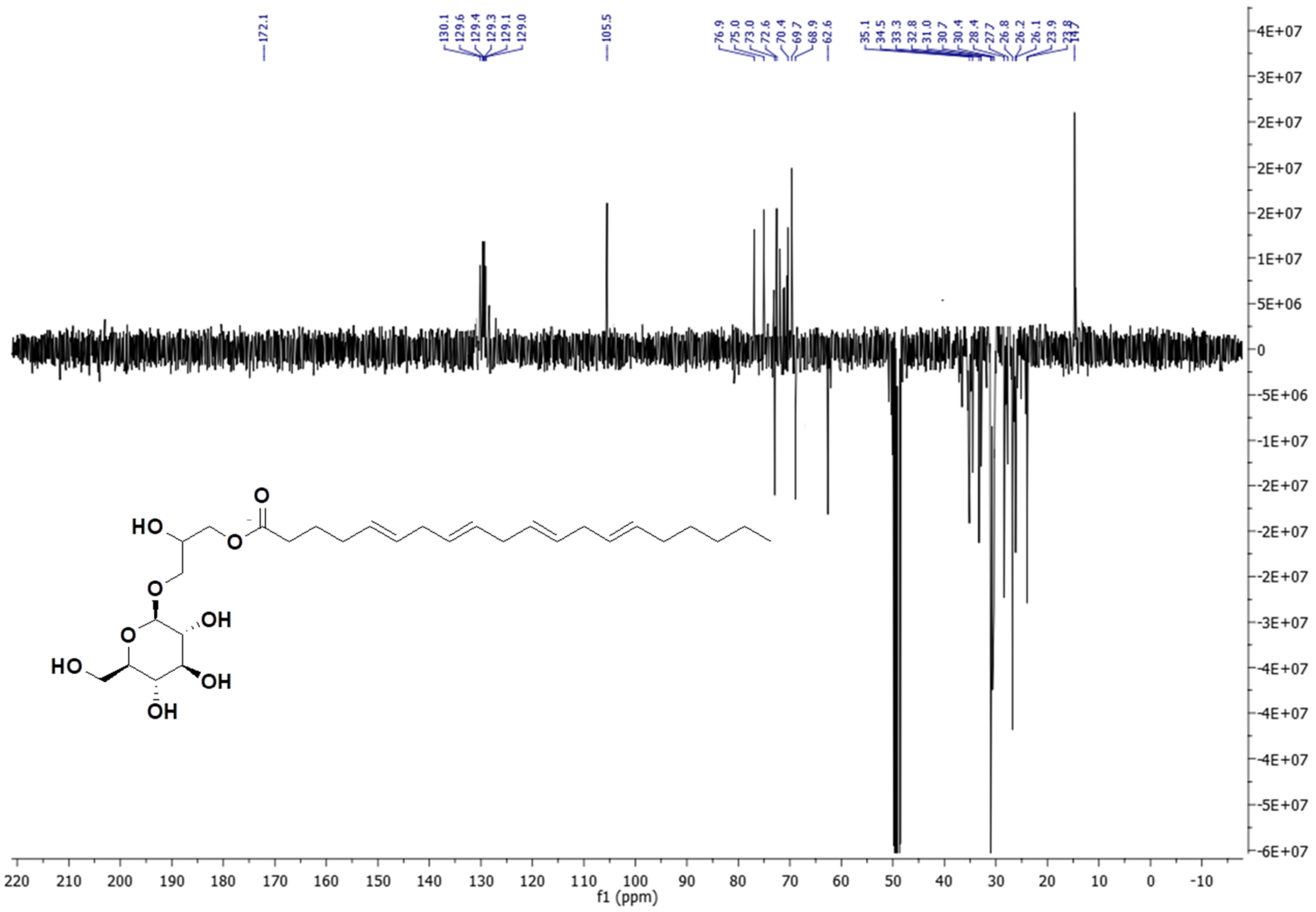

Figure S24. DEPT-Q NMR spectrum of compound **19** measured in CD_3OD-d_6 at 100 MHz

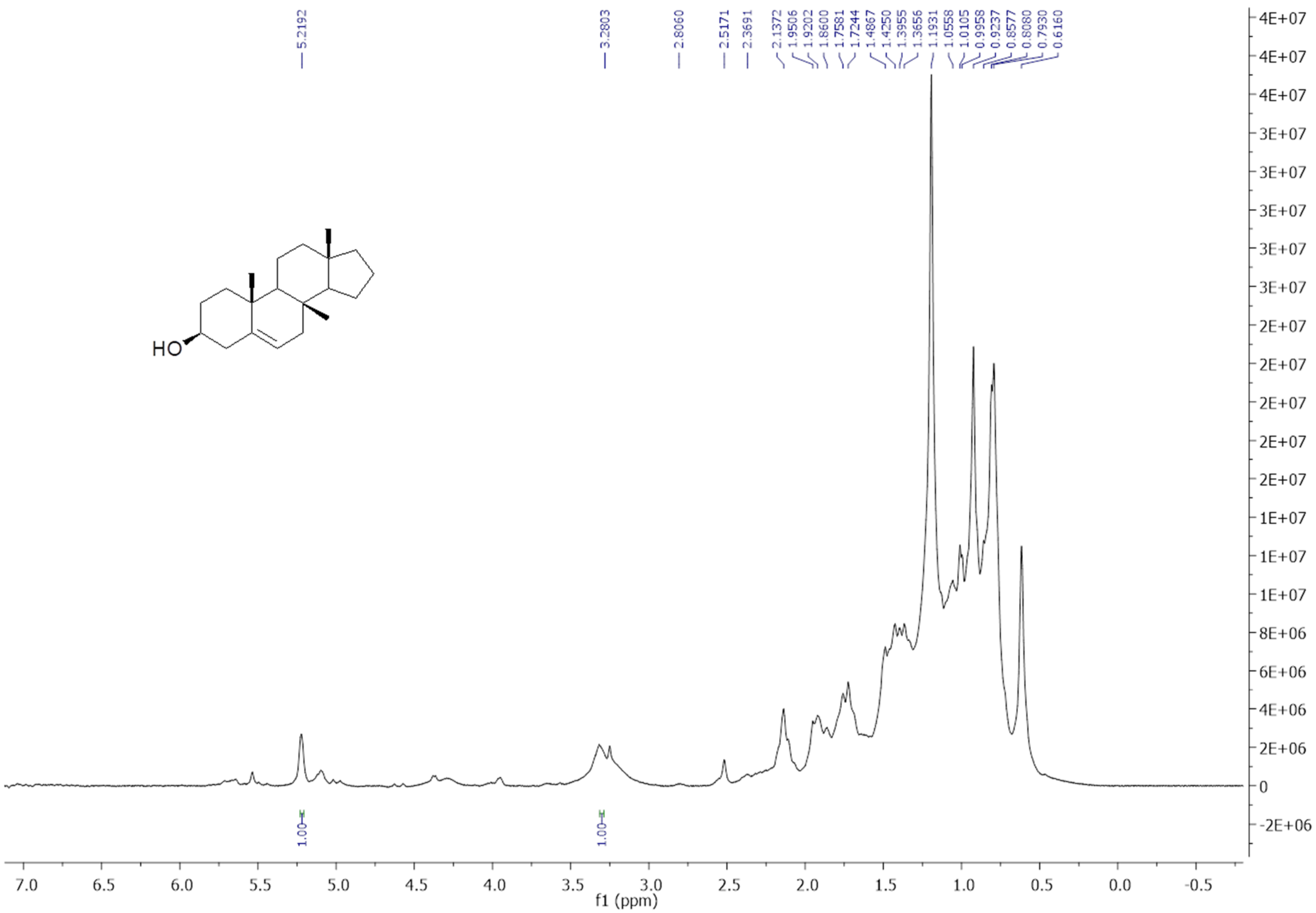

Figure S25. 1H NMR spectrum of compound **20** measured in DMSO-d_6 at 400 MHz

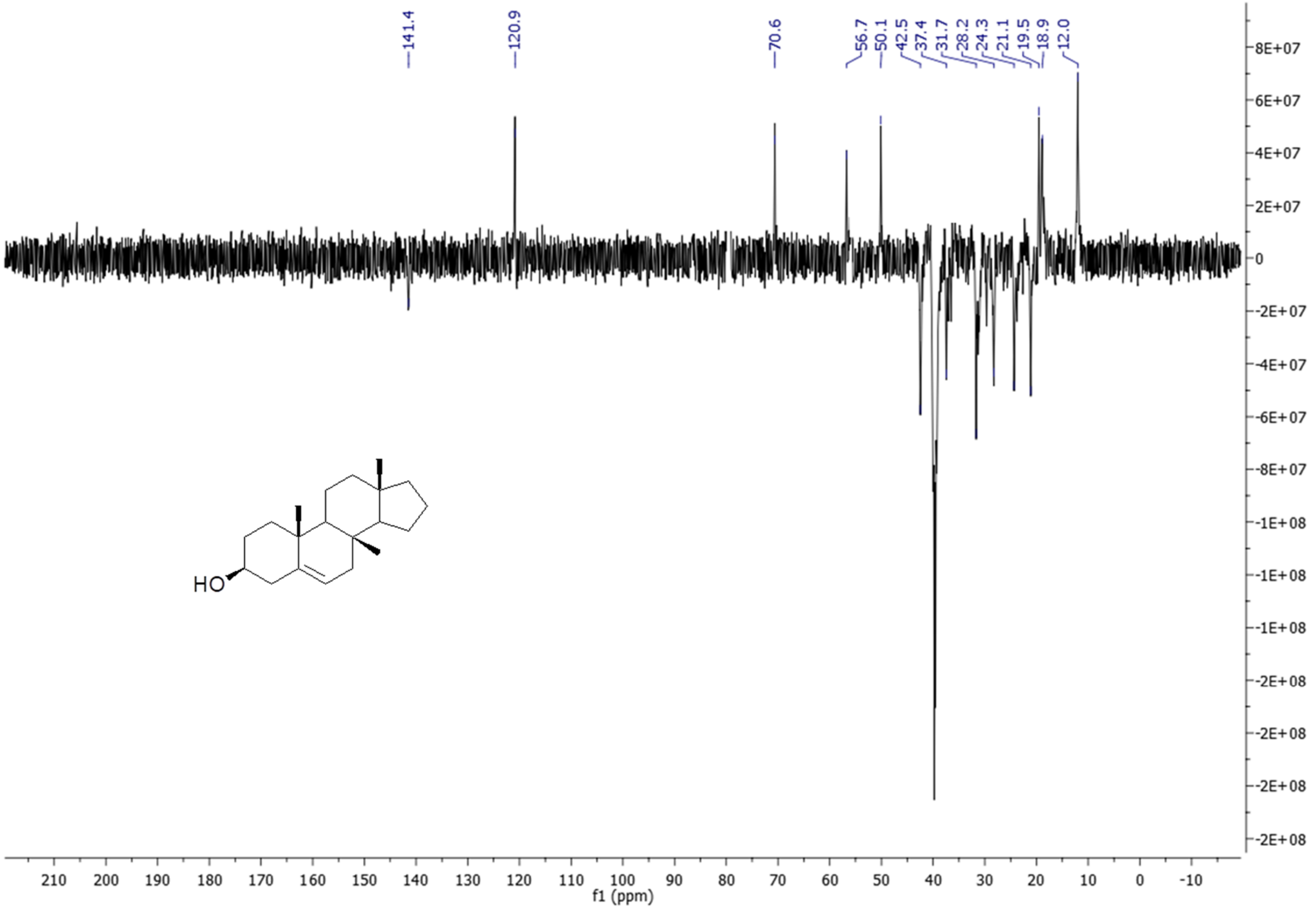

Figure S26. DEPT-Q NMR spectrum of compound **20** measured in DMSO-d_6 at 100 MHz

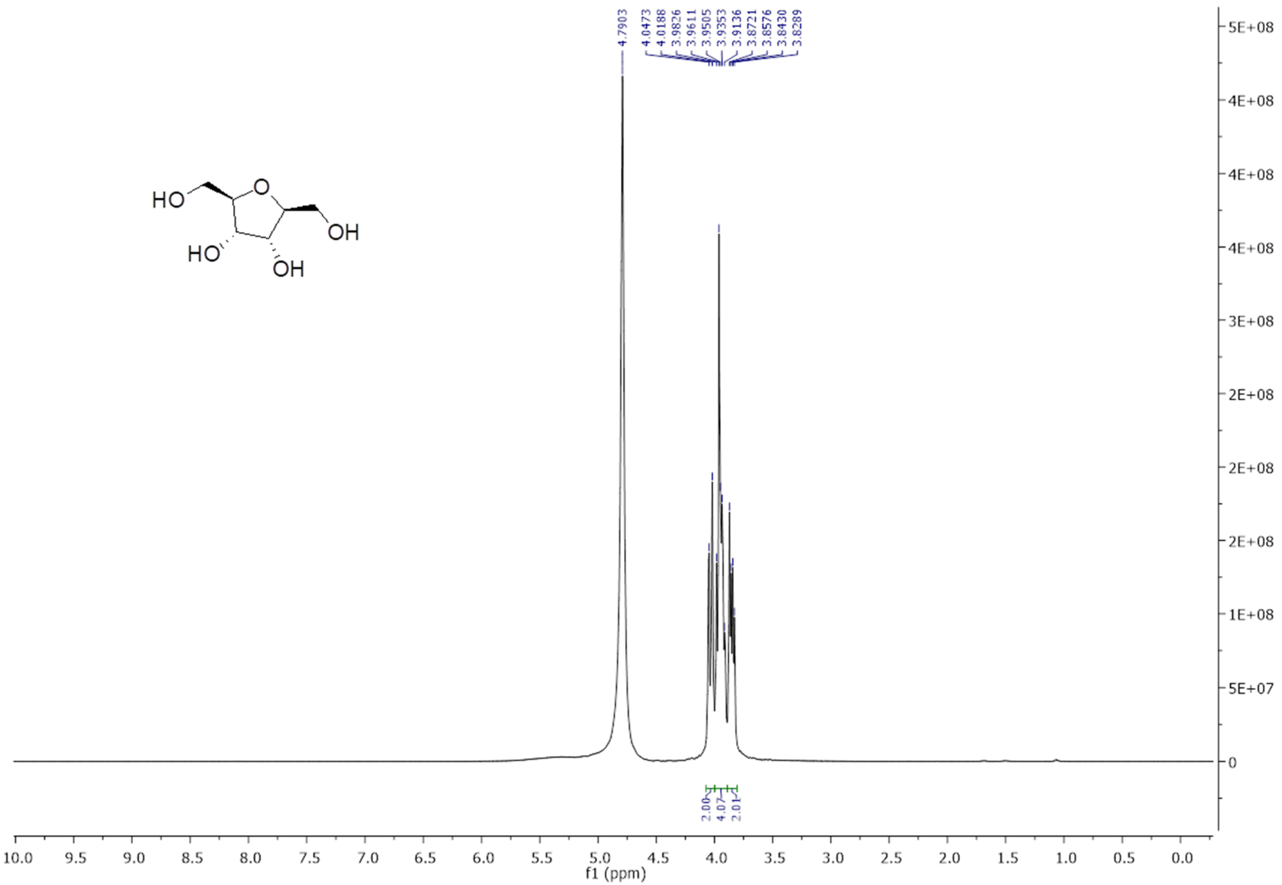

Figure S27. 1H NMR spectrum of compound **21** measured in D_2O at 400 MHz

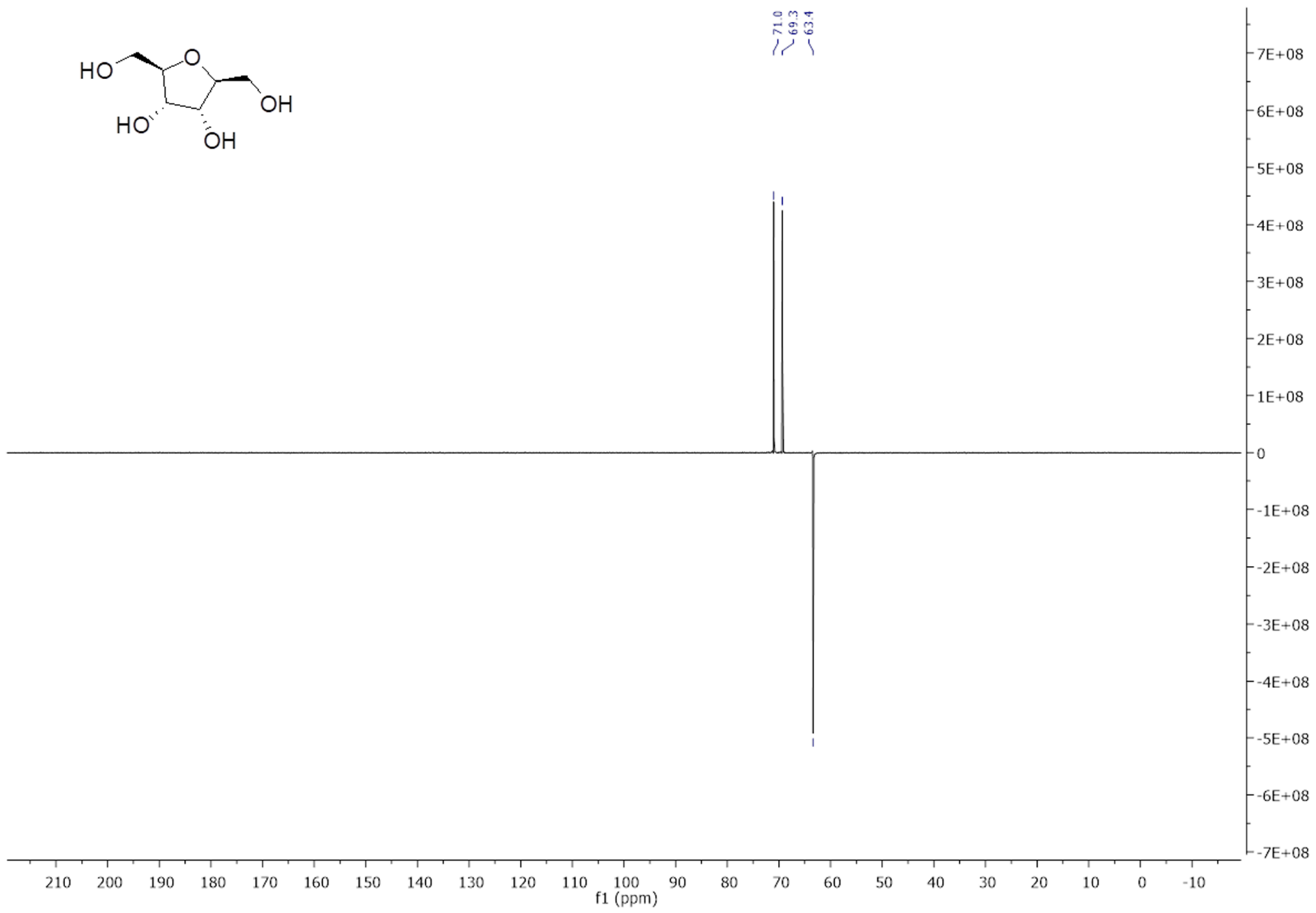

Figure S28. DEPT-Q NMR spectrum of compound **21** measured in D_2O at 100 MHz
